# Serum Vitamin D Levels in Relation to Hypertension and Pre-hypertension in Adults: A Systematic Review and Dose–Response Meta-Analysis of Epidemiologic Studies

**DOI:** 10.3389/fnut.2022.829307

**Published:** 2022-03-10

**Authors:** Elahe Mokhtari, Zahra Hajhashemy, Parvane Saneei

**Affiliations:** ^1^Students' Research Committee, Isfahan University of Medical Sciences, Isfahan, Iran; ^2^Department of Community Nutrition, School of Nutrition and Food Science, Food Security Research Center, Isfahan University of Medical Sciences, Isfahan, Iran

**Keywords:** serum 25-hydroxy vitamin D, hypertension, pre-hypertension, meta-analysis, epidemiologic studies

## Abstract

**Background:**

Findings of observational studies that evaluated the association of serum vitamin D status and high blood pressure were contradictory. This meta-analysis of epidemiologic studies assessed the relation of serum vitamin D levels to hypertension (HTN) and pre-hypertension in adults.

**Methods:**

We conducted a systematic search of all published articles up to March 2021, in four electronic databases (MEDLINE (PubMed), Web of Science (ISI), Embase and Scopus), and Google scholar. Seventy epidemiologic studies (10 prospective cohort, one nested case–control, and 59 cross-sectional investigations) that reported relative risks (RRs), odds ratios (ORs), hazard ratios, or prevalence ratios with 95% CIs for HTN or pre-hypertension in relation to serum vitamin D concentrations in adults were included in the analysis.

**Results:**

In prospective studies, a 16% decrease in risk of hypertension was observed in participants with high levels of serum vitamin D compared to low levels (RR: 0.84; 95%CI: 0.73, 0.96; 12 effect sizes). Dose–response analysis in prospective studies revealed that each 25 nmol/L increase in serum vitamin D concentrations resulted in 5% reduced risk of HTN (RR: 0.95; 95% CI: 0.90, 1.00). Also, a significant nonlinear relationship between serum vitamin D levels and HTN was found (P_nonlinearity_ < 0.001). In cross-sectional investigations, highest vs. lowest level of serum vitamin D was related to reduced odds of HTN (OR: 0.84; 95%CI: 0.79, 0.90; 66 effect sizes) and pre-hypertension (OR: 0.75; 0.95%CI: 0.68, 0.83; 9 effect sizes). Dose–response analysis in these studies showed that each 25 nmol/L increase in serum vitamin D levels was related to a significant 6% reduction in odds of hypertension in all populations (RR: 0.94; 95%CI: 0.90, 0.99) and 3% in studies with representative populations (RR: 0.97; 95%CI: 0.95, 0.99).

**Conclusion:**

This meta-analysis of epidemiologic studies disclosed that serum vitamin D concentrations were inversely related to the risk of HTN in adults, in a dose–response manner in both prospective cohort and cross-sectional studies.

**Systematic Review Registration:** http://www.crd.york.ac.uk/Prospero, identifier: CRD42021251513.

## Introduction

High blood pressure or hypertension (HTN) has a prominent role in cardiovascular disease (CVD). Systolic blood pressure (SBP) ≥ 130 mmHg or diastolic blood pressure (DBP) ≥ 80 mmHg are the threshold values recently proposed to define hypertension (HTN) (1). Hypertension is one of the most important risk factors for global mortality and morbidity and has been associated with non-communicable diseases such as atherosclerosis, cardiomyopathy, and acute myocardial infarction (2). In addition, HTN increases the risk of stroke, heart attack, and kidney failure, which impose a great economic burden on societies (3, 4). The prevalence of HTN varies in different parts of the world and can be influenced by demographic factors such as age, race, gender, and socioeconomic status (5). For example, its prevalence is reported to be 39.1% in Latin America, 29.4% in South Asia, and 22% in Iran (6, 7).

Several modifiable and non-modifiable risk factors, such as age, gender, genetics, high sodium intake, low potassium intake, obesity, lack of physical activity, and unhealthy diet are involved in increasing blood pressure (BP) (8). Previous studies found that serum vitamin D levels could have an inverse association with BP (9). Vitamin D supplementation was also suggested as a critical approach to preventing hypertensive disorders in pregnancy (10). It has been proved that vitamin D insufficiency has a high prevalence worldwide, even in countries that are located in the lower latitude and in industrialized countries where vitamin D fortified foods are easily accessible (11).

Although several epidemiologic studies investigated the association between circulating vitamin D concentrations and hypertension, the findings were contradictory. Some of the research studies documented that lower vitamin D levels significantly increased the risk of hypertension (12, 13), while others did not find a significant association (14, 15). On the other hand, some investigations reported a lower risk of hypertension in vitamin D-deficient individuals; however, these results were not significant (16, 17). In addition, some studies have reported the inverse relationship between vitamin D level and BP only in women (12), or only in male individuals (14). The findings have additionally remained controversial in the case of definitions used to identify hypertensive subjects and vitamin D-deficient/insufficient individuals (14–19). To our knowledge, there is no systematic review and meta-analysis that summarized the relationship between serum vitamin D concentrations and hypertension in observational studies. So, we objected to evaluating the relationship between serum vitamin D concentrations and hypertension/prehypertension in adults and carried out a systematic review and meta-analysis on epidemiologic studies. We also assessed whether serum vitamin D levels could decrease the risk of HTN in a linear or non-linear fashion. We hypothesized that the optimal level of serum vitamin D could be related to a reduced risk of hypertension and prehypertension.

## Methods and Materials

### Search Strategy

We conducted a systematic search of all published articles up to April, 2021, in the electronic MEDLINE (PubMed), Web of Science (ISI), Scopus databases, and Google scholar, with no limitation in language or time of publication. Applied MeSH and non-MeSH keywords in the systematic search are presented in detail in Supplementary Table 1. Furthermore, we performed a manual search in bibliographies of the relevant investigations to identify additional studies. Gray literature, including conference proceedings, unpublished articles, and theses, was not included in the present review. Duplicate studies were removed. Then, two investigators (EM and ZH) independently carried out the article selection by title and abstract screening, and any disagreement was resolved by discussion with the principal investigator (PS) to reach a consensus. The full text of potentially relevant articles was obtained to extract data. We conformed to the Preferred Reporting Items for Systematic Reviews and Meta-Analyses guideline (PRISMA) (20) in the present analysis and the details are presented in Supplementary Table 2. The study protocol was furthermore registered at Prospero (http://www.crd.york.ac.uk/Prospero; no. CRD42021251513).

### Inclusion Criteria

Published studies were included in our analysis if they: (1) were population-based epidemiological studies with cross-sectional, cohort, case-control, or nested case–controls design; (2) conducted on adults ( ≥ 18 years old); (3) considered circulating 25-hydroxy vitamin D levels as the exposure; (4) considered hypertension, high BP, or pre-hypertension as the outcome of interest; 5) reported relative risks (RRs), odds ratios (ORs), hazard ratios (HRs), or prevalence ratios (PRs) and corresponding 95% CIs (or sufficient data for calculating these values) for the association between serum vitamin D levels and hypertension or pre-hypertension.

### Exclusion Criteria

Details of more relevant studies that were excluded are reported in Supplementary Table 3. Studies were excluded if they: (1) considered vitamin D deficiency as the outcome and hypertension as the exposure; (2) reported OR/RR for pregnancy-induced hypertension; (3) considered BP as a continuous outcome; (4) considered hypertension severity as the outcome; (5) provided standard regression (ß) coefficient for the relationship; (6) reported correlation coefficient for the linkage; (7) reported the relationship in children and adolescents. Moreover, all editorials, letters, comments, theses, case-reports, and review articles were not included in our review. In addition, Barcelo et al. reported ORs for two different definitions of hypertension (defined as ≥ 130/85 mmHg vs. ≥ 140/90 mmHg), to avoid overlapping of populations, we used one of these ORs (provided for BP ≥ 130/85 mmHg) (18). Moreover, for those investigations that provided ORs for both vitamin D quartile categories and vitamin D deficiency vs. sufficiency (19), we included only the estimate for vitamin D deficiency vs. sufficiency in the analysis.

### Data Extraction

Based on a pre-designed table, the following data were extracted from each eligible study: the first author's last name, year of publication, study design, duration of follow-up, location, age range or mean age, gender, number of participants, number of hypertensive cases, 25-hydroxyvitamin D [25(OH)D] levels, unit of serum vitamin D, OR, RR, HR or PR, and 95% CI for the association of vitamin D and hypertension or pre-hypertension, methods of serum vitamin D measurement, cut-off points used to define hypertension, the health status of participants, adjustments for potential confounders, and quality scores of studies. Two researchers (EM and ZH) independently extracted data and the principal investigator (PS) supervised the process.

### Quality Assessment of Studies

Newcastle–Ottawa quality assessment scale (NOS) (21) (adapted for cross-sectional and cohort studies) was used to assess the quality of eligible investigations. This scale allocates a total score of 9, as the highest quality, to cohort studies: 4 scores for participant selection (representativeness of the exposed cohort, selection of the non-exposed cohort, and ascertainment of exposure and demonstration that hypertension was not present at the start of the study), 2 scores for comparability (considering controls for the most important factors, including season of blood drawn or sun exposure and additional adjustments for age, gender, and BMI) and 3 scores for outcome assessment (using a validated assessment for hypertension as the outcome, enough follow-up duration for hypertension to incidence and adequate follow-up for cohorts). NOS allocates a total score of 10, as the highest quality, to cross-sectional studies: 5 scores for the selection of participants (representativeness of the sample, sample size satisfaction, explanation for non-respondents, and ascertainment of the exposure assessment), 2 scores for comparability (controlling for the most important factors, including season of blood drawn or sun exposure and additional adjustments for age, gender, and BMI), and 3 scores for outcome assessment (using a validated assessment for hypertension and using an appropriate statistical test). Quality assessment for studies is described in detail in Supplementary Table 4. In this meta-analysis, prospective studies with a score 7 or more and cross-sectional studies with a score of 8 or more were classified as high-quality investigations; those with lower scores were deemed to be low-quality studies. Moreover, Grading of Recommendations, Assessment, Development, and Evaluations (GRADE) (22) was used to determine the quality of evidence through GRADEpro (GRADEproGDT, www.gradepro.org) (23). According to this approach, we examined the main factors that could downgrade the study quality including indirectness of evidence, risk of bias, inconsistency of findings, imprecision of findings, and publication bias. The factors upgrading quality were also included: the evaluation of dose–response analysis, large effect, and plausible confounding. Based on the GRADE approach, the certainty of the body of evidence could be rated in one of four categories: high, moderate, low, and very low. Results of GRADE assessment of the current meta-analysis are presented in Supplementary Tables 5 and 6.

### Statistical Analysis

Reported RR, OR, HR or PR, and 95% CI for the relationship between vitamin D and hypertension (HTN) or pre-hypertension were used to calculate log OR or RR and its standard error. For those studies that reported the estimate for the lowest vs. the highest level of serum vitamin D, we converted the OR to have the estimate for the highest vs. the lowest level. The overall effect size was calculated by using a fixed-effect model when heterogeneity was low (*I*^2^ < 50%) and a random-effect model, that takes between-study variation into account, when heterogeneity was high (*I*^2^ > 50%). We evaluated the between-study heterogeneity through the use of Cochran's Q test and *I*^2^. In cases of significant between-study heterogeneity, we used subgroup analysis based on confounders/moderators (such as study location [Asian vs. non-Asian countries], developmental status, gender, levels of vitamin D used for comparison, cut-off points used to define hypertension, the health status of participants, adjustment for the season of blood drawn or sun exposure, additional adjustment for age, and gender and BMI, representativeness of the population, and quality of studies) to explore possible sources of heterogeneity. Between-subgroup heterogeneity was examined through a fixed-effect model. Sensitivity analysis was done to examine the extent to which inferences might depend on a particular study. Publication bias was assessed by visual inspection of funnel plots and formal statistical assessment of funnel plot asymmetry was performed by Begg's test and Egger's regression asymmetry test.

For dose–response analysis, a previously described method by Greenland and Longnecker (24) and Orsini et al. (25) was used. The natural logs of the ORs, RRs, HRs or PRs and 95% CIs across categories of serum vitamin D were used to compute study-specific slopes (linear trends) and 95%CIs for 25 nmol/L (or 10 ng/mL) which is the difference between severe deficiency (<25 nmol/L), deficiency (25–50 nmol/L), insufficiency (50–75 nmol/L), and sufficiency levels (>75 nmol/L) of serum vitamin D. In this method, the distribution of individuals with hypertension and the OR/RR/HR/PR with the variance estimates for at least three quantitative categories of exposure for non-linear trends were required. The mean or median level of serum vitamin D in each category was assigned to the corresponding OR/RR/HR/PR for each study. For studies that reported the serum 25(OH) D levels as ranges, we estimated the midpoint in each category by calculating the average of the lower and upper bounds. When the highest category was open-ended, the length of the open-ended interval was assumed to be the same as that of the adjacent interval. When the lowest category was open-ended, the lower boundary for 25(OH) D was set to zero. Restricted cubic splines (3 knots at fixed percentiles of 10, 50, and 90% of the distribution) were used to examine potential non-linear dose–response associations between serum vitamin D and risk of hypertension. Statistical analyses were done with STATA version 14.0 (STATA Corp, College Station, TX, United States). All STATA codes used in the analyses are presented in Supplementary Table 7. *P*-values < 0.05 were considered statistically significant for all tests including Cochran's *Q*-test.

## Results

**Figure 1:**
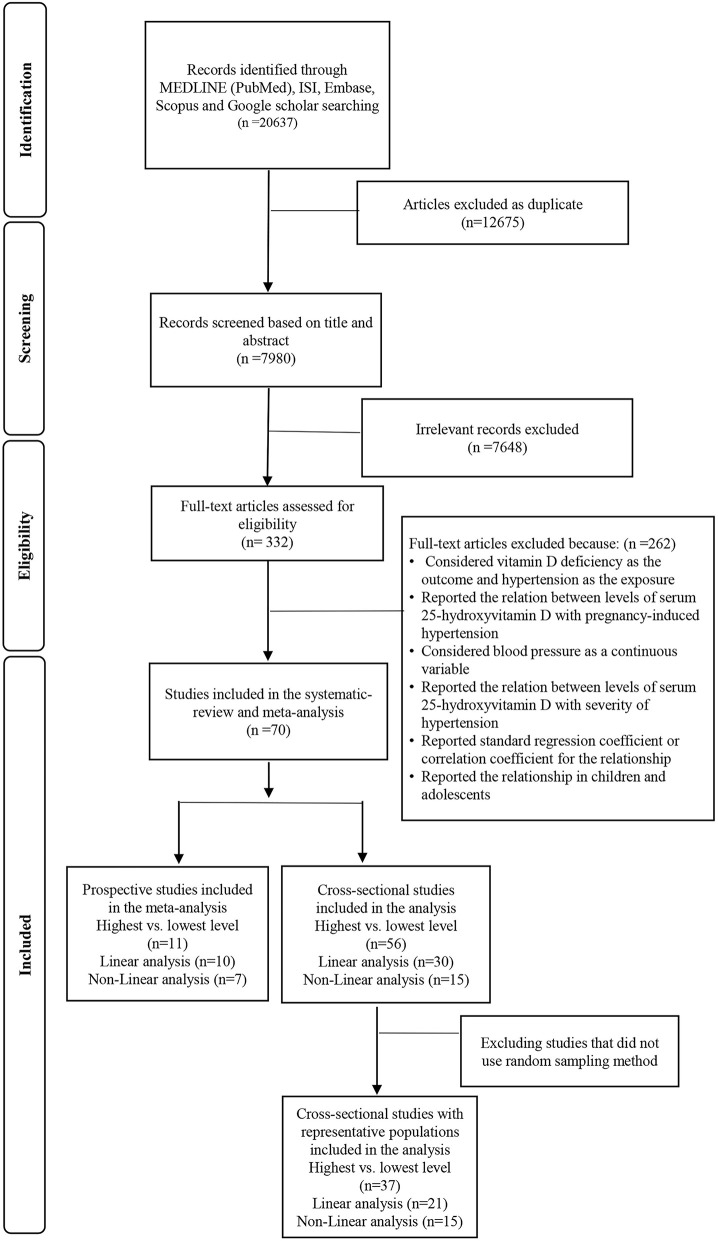
Flowchart of the study selection process.

In total, our initial systematic search resulted in 4,255 articles after excluding duplicate studies. In the first round of screening, the titles and abstracts were separately screened. Then, the full text of 102 studies was assessed in the second round. Finally, 70 studies were eligible to be included in the systematic review and meta-analysis. Details of flow diagram search strategy and study selection are presented in Figure 1.

### Study Characteristics

Details of 70 eligible studies that were included in this systematic review and meta-analysis are summarized in Table 1. These investigations were published between 2005 and 2020. A total of 59 of the investigations had a cross-sectional design (13–19, 26–38, 41–43, 45, 47–50, 52–56, 60–75, 77–86), one investigation was a nested case–control (12), and 10 others were cohorts (39, 40, 44, 46, 51, 57–59, 76, 87). One of these cohorts (46) had reported the association in both baseline and follow-up; so, it was included in the analysis of both cross-sectional and cohort studies. Overall 66,757 and 260,944 participants were, respectively, included in cohort and cross-sectional studies. Among cross-sectional articles, fourteen studies were carried out in the United States (16, 29, 32, 36, 38, 53, 60, 63, 64, 67, 68, 77, 78, 82), twelve in South Korea (13, 26, 28, 33, 47, 48, 55, 70, 83–86), nine in China (15, 41, 50, 52, 54, 72–75), four in Iran (30, 37, 45, 65), two in the Netherlands (17, 27), Australia (42, 71), Thailand (62, 69), Mexico (19, 66) and the remaining in Brazil (31), India (34), Japan (35), Spain (18), Sweden (14), United Kingdom (43), Canada (49), Israel (56), Jordon (61), France (79), and Italy (81); the last study was a multi-country investigation (80). In the case of cohort studies, five cohorts were carried out in the United States (39, 40, 51, 58, 59) and the remaining in Norway (44), Finland (46), Netherlands (57), Australia (87), and Denmark (76). Also, the only nested case–control study was done in the United States (12). Four studies were performed on men, 10 investigations on women, eleven others on men and women separately, and the last forty-five publications on both genders. Different methods were used to measure serum vitamin D concentrations including radioimmunoassay (RIA) (*n* = 22 studies), chemiluminescence immunoassay (CLIA) (*n* = 19), electrochemiluminescence immunoassay (ECLIA) (*n* = 6), ELISA (*n* = 6), enzyme immunoassay (EIA) (*n* = 4), competitive protein binding assay (CPBA) (*n* = 2), high-performance liquid chromatography (HPLC) (*n* = 2), and other assays (*n* = 6); while three other studies did not mention a particular method for vitamin D measurement. In the case of outcomes of interest, four studies reported the association between vitamin D levels and pre-hypertension (defined as BP: 120–139/80–89 mmHg) (63, 64, 77, 82). Thirty-two other studies assessed the association with hypertension (defined as BP ≥ 140/90 mmHg) and thirty-one others used the cut-off point of 130/85 mmHg to define hypertension and three other investigations evaluated the association with both hypertension ( ≥ 140/90 mmHg) and pre-hypertension in their population (15, 67, 68). While most of the investigations were conducted on healthy participants (*n* = 59), the participants of 10 other research studies were done on populations with prostate, lung, colon, and ovarian cancers (38), colorectal neoplasia (32), sleep apnea (18), hemodialysis (37), systemic lupus erythematous (19, 49), peritoneal dialysis (73), and obesity (80, 81) and the last study was done on elderly inpatients (79). Also, one study (65) reported ORs for both metabolically healthy obese individuals and metabolically unhealthy obese individuals. The most adjusted confounders in the studies were age (*n* = 64), gender (*n* = 53), BMI (*n* = 45), physical activity (*n* = 42), smoking status (*n* = 42), alcohol (*n* = 36), and season of blood drown (*n* = 28). It is worth noting that none of the cohort studies had controlled the baseline vitamin D levels in their analysis. Among cohorts, the NOS scores were between 6 and 9. Eight studies were of high quality, while 2 others were classified as of low quality. Also, the only nested case–control had low quality. Four prospective studies (36%) were judged to have low quality, due to the non-representativeness of the exposed cohort. Two prospective studies (18%) were judged to have low quality arising from the demonstration that the outcome of interest was not present at the start of the study. Two prospective studies (18%) were judged to have low quality because of not having adequate follow-up. Among cross-sectional investigations, the NOS scores ranged between 4 and 10; 33 studies had high quality and 26 others had low quality. Nineteen cross-sectional studies (32%) were judged to have low quality due to non-representativeness of the sample. Twenty-eight cross-sectional studies (47%) were judged to have low quality arising from small sample size. Fourteen cross-sectional studies (24%) were judged to have low quality because they did not report details of non-respondents. Sixteen cross-sectional studies (27%) were judged to have low quality due to no appropriate comparability of subjects in different outcome groups (Supplementary Table 4).

**Table 1:** Main characteristics of included cohort and cross-sectional studies examined the association between serum vitamin D levels and high blood pressure in adults.

	**First author (year)**	**Study design/ name study**	**Country Latitude, **°**N**	**Age range/ mean age**	**Gender**	**No. of Participants**	**Hypertensive Cases**	**25 (OH)D Levels, unit**	**OR/RR (95% CI)**	**Method (Exposure)**	**Definition (Outcome)**	**Subject**	**Adjustment^a^**	**Quality of studies**
1	Lee et al. (26)	Cross-sectional (Baseline Elderly cohort)	Korea	65 ≤	Both	2,936				CLIA	SBP≥ 130	Elderly	1, 5, 6, 7,10, 11, 18	8
					Men	987		Q1 (4.20–14.19) ng/ml	1.07 (0.69, 1.67)		DBP≥ 85 mmHg			
								Q2 (14.20–18.99)	1.12 (0.73, 1.72)					
								Q3 (19.00–24.19)	1.05 (0.69, 1.59)					
								Q4 (24.20–51.90)	1.00 (ref.)					
					Women	1,949								
								Q1 (4.10–11.19)	1.24 (0.92, 1.66)					
								Q2 (11.20–15.59)	1.24 (0.93, 1.64)					
								Q3 (15.60–21.59)	1.24 (0.93, 1.64)					
								Q4 (21.60–54.90)	1.00 (ref.)					
2	Vitezova et al. (27)	Cross-sectional (Baseline Cohort	Chile	55 ≤	Both	3,240				ECLIA	SBP≥ 130 DBP≥ 85 mmHg	Middle-aged and elderly adults	1, 2, 5, 7, 8, 9, 11, 16, 33, 37, 41, 42	9
		Rotterdam Study1989–1993)	Rotterdam, Netherlands			1,833		T1 (<50) nmol/l	1.00 (ref.)					
						874		T2 (50–75)	1.04 (0.85, 1.27)					
						533		T3 (≥75)	0.89 (0.70, 1.12)					
														
														
3	Chon et al. (28)	Cross-sectional (Baseline Cohort	South Korea	≥18	Women	4,364				ECLIA	SBP≥130	Post-menopausal	1, 5, 6, 7, 8, 11, 14, 59	9
		KNHANE)				1,445		T1 (3.07–14.89) ng/ml	1.00 (ref.)		DBP≥ 85 mmHg	women		
						1,445		T2 (14.9–20.96)	0.95 (0.81, 1.12)					
						1,446		T3 (20.97–66.96)	0.83 (0.71, 0.98)					
4	Maki et al. (29)	Cross-sectional (Baseline Cohort	U.S	20 ≤	Both	3,529				RIA	SBP≥130	Non- institutionalized civilian U.S. population	1, 2, 3, 5, 6, 7, 8, 16, 20, 32, 40, 43	9
		(NHANES)						Q1 (7.5–44.9) nmol/l	1.00 (ref.)		DBP≥ 85 mmHg			
								(45–59.9) Q2	0.77 (0.58, 1.01)		or taking anti			
								(60–74.9) Q3	0.74 (0.55, 1.00)		hypertensive			
								(75–215) Q4	0.71 (0.49, 1.02)		medication			
5	Mansouri et al. (30)	Cross-sectional	Tehran, Iran	35 ≤	Both	352				ELISA	SBP≥130	High educated population	1, 2, 5, 7, 12, 31, 34	7
						89		Q1	1.00 (ref.)		DBP≥ 85 mmHg			
						90		Q2	0.42 (0.18, 1.01)					
						85		Q3	0.38 (0.16, 0.92)					
						88		Q4	0.45 (0.19, 1.03)					
6	Schmitt et al. (31)	Cross-sectional cohort study	São Paulo, Brazil	45–75	Women	463				CLIA	SBP≥130	Post-menopausal women	1, 4, 5, 7, 60	6
							219	<30 ng/ml	1.23 (0.79, 2.85)		DBP≥ 85 mmHg			
							98	≥30	1.00 (ref.)					
7	Bea et al. (32)	Cross-sectional	USA	65.7 ± 8.75	Both	2,096				CLIA	SBP≥ 130	Colorectal neoplasia patients after surgery	1, 2, 3, 22, 61	7
					Men		117	<20 ng/ml	1.00 (ref.)		DBP≥ 85			
							315	20– 30	0.72 (0.51, 1.03)		mmHg			
							334	≥30	0.72 (0.51, 1.03)					
														
														
					Women		116	<20 ng/ml	1.00 (ref.)					
							136	20– 30	1.02 (0.70, 1.49)					
							72	≥30	1.13 (0.71, 1.78)					
8	Kim and Kim (33)	Cross-sectional	South Korea	≥19	Both	5,559				RIA or EIA	SBP≥130 and/or	Adults	1, 2, 4, 5, 6, 7, 8, 9, 11, 15, 21	10
			latitude 33–38N			1,112		Q1 (2.65–12.87) ng/ml	1.00 (ref.)		DBP≥ 85mmHg		.	
						1,112		Q2 (12.88–16.87)	0.93 (0.72, 1.33)		or currently undergoing treatment for			
						1,112		Q3 (16.88–20.8)	1.18 (0.92, 1.53)		hypertension			
						1,112		(20.82–26.13) Q4	0.80 (0.61, 1.03)					
						1,111		Q5 (26.14–58.66)	1.05 (0.79,1.42)					
9	Majumdar et al. (34)	Cross-sectional	India	18–75/	Both	441				EIA	SBP≥130	Adults	1, 4, 5, 16	5
				39 ± 12.8	Men	237		Q1 (<28.2) nmol/l	1.00 (ref.)		DBP≥ 85 mmHg			
								Q2 (2.82–38)	1.40 (0.50, 3.30)					
								Q3 (38.1–47)	1.10 (0.50, 2.80)					
								Q4 (47.1–57.8)	0.90 (0.30, 2.10)					
								Q5 (>57.8)	0.50 (0.20, 1.30)					
														
					women	204		Q1 (<25.2)	1.00 (ref.)					
								Q2 (25.2–34.2)	2.30 (0.70, 7.20)					
								Q3 (34.3–42.9)	2.00 (0.60, 6.30)					
								Q4 (43–53.5)	1.50 (0.40, 5.10)					
								Q5 (>53.5)	2.20 (0.70, 7.10)					
10	Akter et al. (35)	Cross-sectional	Japan	18–69	Both	1,790				CPBA	SBP≥130	Workers	1, 2, 4, 5, 6, 7, 22,62	7
						730		<20 ng/ml	1.00 (ref.)		DBP≥ 85 mmHg			
						921		20–30	1.04 (0.83, 1.32)					
						139		30 ≤	0.92 (0.60, 1.40)					
11	Barceló et al. (18)	Cross-sectional	Bunyola, Spain	52.25 ± 12.4	Both	826				EIA*	SBP≥130 or	Newly diagnosed with obstructive sleep apnea	1, 2, 11, 16	4
	2013					105		≥15 ng/ml	1.00 (ref.)		DBP≥ 85 mmHg			
						377		16–30	0.97 (0.51, 1.85)					
						344		>30	0.76 (0.39, 1.49)					
12	Mitri et al. (36)	Cross-sectional	USA	25 ≤	Both	2,000				Liquid chromato- graphy, tandem mass spectrometry	SBP≥130 and/or	Diabetes high-risk population	1, 2, 3, 4, 5, 6, 7, 11, 19, 43, 63, 64	9
						666		T_1_ 12.1 (9.7–14.3) ng/ml	1.00 (ref.)		DBP≥ 85 mmHg or			
						667		T_2_ 20.3 (18.3–22.7)	1.01 (0.88, 1.16)		current antihypertensive drug treatment			
						667		T_3_ 30.6 (27.5–34.9)	1.03 (0.76, 1.41)					
														
13	Ahmadi et al. (37)	Cross-sectional	Tehran, Iran	24 −94/	Both	145				EIA	SBP≥130	Hemodialysis patients	1, 2	5
				58. ± 16.0		27		≤ 15 ng/ml	1.05 (0.44, 2.49)		or			
						76		16–30	1.11 (0.48, 2.57)		DBP≥ 85 mmHg or treatment of previously			
						42		>30	1.00 (ref.)		diagnosed hypertension			
14	Brock et al. (38)	Cross-sectional	USA	63 ± 5	Both	2,465		<37 nmol/l	1.00 (ref.)	Radio-iodinated tracer assay	Self-reported	Middle-aged Caucasian from Prostate, Lung Colon and Ovarian Cancer Screening Trial	2, 4, 5, 7, 8, 11, 19, 20, 22, 66, 67, 68	7
								37–50	0.90 (0.60, 1.20)					
								50–80	0.90 (0.70, 1.20)					
								80≥	1.00 (0.70, 1.40)					
														
15	Burgaz et al. (14)	Cross-sectional	Uppsala, central Sweden	0.6 ± 71	Men	35		<37.5 nmol/l	3.3 (1.0, 11.0)	HPLC	SBP>130	Elderly men	4, 6, 7, 11	8
		(Uppsala Longitudinal Study of Adult Men (ULSAM)				798		≥ 37.5	1.00 (ref.)		and/or DBP>85 mmHg using 24–h BP measurements			
														
														
16	Dorjgochoo et al. (15)	Cross-sectional	Shanghai, China	40– 74	Both	1,460				CLIA	HTN: SBP ≥140	Adults	1, 2, 4, 5, 6, 7, 8, 11, 14, 19, 20, 30, 36	9
		(from two large, population-based, prospective cohort studies			Men	405	193	Deficient (37.5> nmol/l)	1·00 (ref.)		or			
		Women's Health Study (SWHS)						Insufficient (37.5– 74.9)	0·56 (0·26–, 1·21)		DBP ≥90mmHg			
		And						Sufficient ( ≤ 75.0)	0·42 (0·12, 1·43)					
		Men's Health Study (SMHS))												
					Women	1,055	354	Deficient (37.5>)	1·00 (ref.)					
								Insufficient (37.5– 74.9)	1·09 (0·73, 1·63)					
								Sufficient (≥75.0)	1·07 (0·31, 3·72)					
											Pre-HTN:			
					Men	405	155	Deficient (37.5>)	1·00 (ref.)		SBP: 120–139 mmHg and/or DBP: 80–89 mmHg			
								Insufficient (37.5– 74.9)	0.57 (0.26, 1.22)					
								Sufficient ( ≤ 75.0)	0.46 (0.14, 1.56)					
														
					Women	1,055	452	Deficient (37.5>)	1·00 (ref.)					
								Insufficient (37.5– 74.9)	1.11 (0.77, 1.60)					
								Sufficient (≥75.0)	1.51 (0.49, 4.60)					
17	Forman et al. (12)	Nested case-control study (NHS2)	USA	43 (40– 46)	Women	1,484			1.47 (1.10, 1.97)	EIA	Self-reported	Adults	1, 3, 4, 7, 11, 18, 36, 44, 46, 51, 69, 70, 71, 72	7
		/year of follow-up: 8–10 years)				975		<30.0 ng/mL	1.00 (ref.)					
						509		≥ 30.0						
														
18	Anderson et al. (39)	Cohort	Salt Lake City, Utah, USA	21 ± 55	Both	41,504			HR	CLIA	NR	Adults	1, 2, 58, 89, 131	6
		1.3 years (maximum 9.3)				6,909		(deficient) ≤ 15 ng/ml	1.62 (1.38, 1.89)					
						19,474		(insufficient) 16–30	1.18 (1.05, 1.33)					
						15,121		normal >30	1.00 (ref.)					
19	Forman et al. (40)	Cohort	Boston,	64.5	Both					RIA	SBP>	Adults	1, 3, 4, 7, 73	7
		(4–8 y)			Men	613		<15 ng/ml	6.13 (1.00, 37.8)		140 and DBP> 90 mm Hg	.		
		(Based on NHS,HPFS)						15– 29	1.12 (0.51, 2.48)					
								≥ 30	1.00 (ref.)					
														
					Women	1,198		<15	2.67 (1.05, 6.79)					
								15–29	0.85 (0.53, 1.34)					
								≥ 30	1.00 (ref.)					
20	García-Carrasco et al. (19)	Cross-sectional	Puebla, México	43.3 ± 11.8	Women	160		<20 ng/ml	1.00 (ref.)	CLIA	SBP≥ 135 or	Non-diabetic SLE women	1, 4, 5	6
								20–30	0.40 (0.10, 1.20)		DBP≥ 85 mm Hg or taking			
								≥ 30	0.43 (0.10, 3.80)		medication for hypertension;			
21	Hidru et al. (41)	Cross-sectional	Dalian, China	62.02 ± 5.73	Both	2,624				ECLIA	SBP>140	Middle-aged and Elderly Chinese Population	1, 2, 4, 5, 6, 7, 44, 53, 54,55, 56, 58	7
		(PCSRFHFEP prospective cohort)			Men	1,105		≤ 12.04 nmol/l	1.00 (ref.)		DBP >90 mmHg			
								12.05–16.50	1.09 (0.76, 1.56)		Or self-reported history of hypertension with the current use of blood pressure-reducing medication			
								16.51–22.69	1.40 (0.97, 2.02)					
								≥22.70	1.07 (0.74, 1.54)					
														
					Women	1,519		≤ 8.60	1.00 (ref.)					
								8.61–12.30	0.84 (0.62, 1.14)					
								12.31–16.83	0.76 (0.56, 1.03)					
								≥16.84	0.86 (0.63, 1.17)					
22	Hirani et al. (42)	Cross-sectional	Sydney, Australia	70 ≤	Men	1,659		SBP ≥140 mmHg (reference <140 mmHg)		RIA	SBP≥ 140 mmHg	Community-dwelling men aged 70 and	Not adjusted because not associated with 25 (OH)D.	7
								<50.0 nmol/L				older		
								50.0– 74.9	0.81 (0.60, 1.09)		DBP ≥ 90 mmHg			
								≥75.0	1.03 (0.76, 1.39)					
									1.00 (ref.)					
								DBP ≥90 mmHg (reference <90 mmHg)						
								<50.0						
								50.0– 74.9						
								≥75.0	1.00 (0.69, 1.44)					
									1.07 (0.74, 1.55)					
									1.00 (ref.)					
23	Hypponen et al. (43)	Cross-sectional	London, U.K	45 (44–46)	Both	6,293		9–45 nmol/l	1.00 (ref.)	automated IDS OCTEIA ELISA	SBP>140	Adults	2, 4, 5, 6, 7, 11, 17, 74, 75, 76	10
		Based on 1958						46–67	0.80 (0.68, 0.94)		DBP >90 mmHg or use of antihypertensive medication			
		British Birth cohort						68–231	0.72 (0.61, 0.86)					
24	Jorde et al. (44)	Longitudinal.	Tromsø, Norway	56.2 ± 9.3	Both	2,385				ECLIA	SBP≥ 140 and/or DBP≥ 90 mm Hg	Adults	1, 2, 4, 7	7
		(1994–2008)				532		<41.4 nmol/l	1.01 (0.78, 1.32)					
						599		41.4–51.5	1.06 (0.83, 1.37)					
						625		51.6–62.6	1.12 (0.87, 1.43)					
						629		>62.6	1.00 (ref.)					
25	Joukar et al. (45)	Cross-sectional (The PERSIAN Guilan Cohort Study (PGCS))	Guilan	70–35	Both	9,520				ECLIA	SBP≥140 and/or	Adults	1, 2, 4, 5, 6, 7, 8, 15,17, 20, 44, 77	9
			Iran			1,957		<30 nmol/l	1.00 (ref.)		DBP≥ 90 mmHg and/or taking antihypertensive drugs			
						2,762		30 −50	1.10 (0.94, 1.27)					
						4,801		≥50	1.09 (0.94, 1.25)					
26	Ke et al. (46)	Cross-sectional	Finland	50–69	Men	2,271				RIA , with ELISA and CLIA in a sub-set of study	SBP≥ 140 or DBP	Male smoker	1, 4, 6, 8, 11, 19, 22, 78, 79, 80	6
						505		≤ 25 nmol/l	1.00 (ref.)		≥ 90 mm Hg			
						517		25–37	1.00 (0.80, 1.10)					
						541		37–50	0.80 (0.60, 1.00)					
						560		50–80	0.80 (0.60, 1.00)					
						148		≥80	0.90 (0.60, 1.10)					
27	Ke et al. (46)	Cohort/4 years of followup	Finland	50–69	Men	1,957				RIA , with ELISA and CLIA in a sub-set of study	SBP≥ 140 or DBP	Male smoker	1, 4, 6, 8, 11, 19, 22, 78, 79, 80	8
								≤ 25 nmol/l	1.00 (ref.)		≥ 90 mm Hg			
								25–37	1.20 (0.90, 1.60)					
								37–50	1.00 (0.70, 1.30)					
								50–80	0.90 (0.70, 1.20)					
								≥80	1.00 (0.60, 1.50)					
28	Kim and Kim (33)	Cross-sectional	South Korea	19 ≤	Both	20,440				RIA	SBP ≥ 140	Adults	1, 4, 5, 6, 7, 8,15, 22, 23, 24, 81	10
		(based on the fourth and fifth KNHANES)			Men						or			
				(19–64)		1,557		Q1 (7.5–35.6 nmol/l)	1.00 (ref.)		DBP≥ 90mm Hg, or the current use of anti-hypertensive medication			
						1,558		Q2 (35.7–44.6)	0.82 (0.66, 1.02)					
						1,553		Q3 (44.6–55.0)	0.90 (0.73, 1.10)					
						1,554		Q4 (55.1–150.8)	0.87 (0.70, 1.08)					
														
				(≥65)		519		Q1 (10.8–38.7)	1.00 (ref.)					
						520		Q2 (38.8–49.4)	1.21 (0.87, 1.69)					
						516		Q3 (49.4–61.9)	1.08 (0.77, 1.51)					
						518		Q4 (62.0–134.0)	0.89 (0.64, 1.22)					
														
				(19–64)	Women	2,363		Q1 (7.4–30.8)	1.00 (ref.)					
						2,360		Q2 (30.8–38.6)	0.93 (0.75, 1.15)					
						2,368		Q3 (38.7–48.3)	0.83 (0.65, 1.06)					
						2,355		Q4 (48.3–137.3)	0.73 (0.58, 0.91)					
														
				(≥65)		676		Q1 (10.3–33.6)	1.00 (ref.)					
						674		Q2 (33.7–43.7)	0.77 (0.58, 1.03)					
						676		Q3 (43.7–55.8)	0.88 (0.65, 1.18)					
						673		Q4 (55.8–167.1)	0.80 (0.61, 1.06)					
29	Kim and Kim (33)	Cross-sectional	South Korea	≥50	Both	5,260				RIA	SBP≥ 140	Middled-aged and Older Korean Adults	1, 2, 4, 5, 6, 7, 8, 15, 18, 22, 23, 24, 45	10
		(based on the fifth KNHANES (V−1,2))				1,315		Q1 (10.3–35.5 nmol/l)	1.00 (ref.)		DBP ≥ 90 mm Hg, or current use of antihypertensive medication			
						1,316		Q2 (35.5–45.2)	0.83 (0.67, 1.03)					
						1,313		Q3 (45.2–57.3)	0.85 (0.68, 1.05)					
						1,316		Q4 (57.3–133.6)	0.86 (0.70, 1.07)					
30	Kim (47)	Cross-sectional	South Korea	≥50	Both	2,624				RIA	SBP≥ 135	Middled-aged and Older Korean Adults	1, 2, 4, 5, 6 ,7, 8, 9, 15	10
		(Based on fifth KNHANES (V−1))				656		Q1 (10.3–35.6 nmol/l)	1.00 (ref.)		or DBP≥ 85 mm Hg or daily use of antihypertensive medication			
						654		Q2 (35.6–45.9)	0.91 (0.71, 1.16)					
						657		Q3 (45.9–59.2)	0.92 (0.72, 1.18)					
						657		Q4 (59.2–122.6)	0.76 (0.59, 0.97)					
31	Kim (47)	Cross-sectional	Seoul	≥30	Both	379				RIA	SBP≥ 135	North Korean refugee health in South Korea	1, 2, 4, 5, 7, 11, 82	7
		( the longitudinal	South Korea			36		<10 ng/mL	2.69 (0.58, 12.60)		or DBP≥ 85 mm Hg or treatment for hypertension			
		Cohort NORNS)				294		20–Oct	2.94 (0.88, 9.88)					
		2008–2012				49		20–30	1.00 (ref.)					
32	Kim et al. (13)	Cross-sectional	Chungju, Korea	≥40	Both	324		Q1 (10.0–29.7 nmol/l)	1.00 (ref.)	Chemilumine scence assays	SBP≥ 135	Middled-aged and Older Korean Adults	1, 2, 4, 5, 6, 7, 11, 18, 19, 22, 24 ,46	10
				(median age 65.8 years)				Q2 (30.0–39.2)	0.62 (0.38, 1.01)		or DBP≥ 85 mmHg or on antihypertensive drug treatment			
								Q3 (39.4–49.4)	0.94 (0.55, 1.61)					
								Q4 (49.7–61.2)	0.84 (0.48, 1.48)					
								Q5 (61.4–116.8)	0.47 (0.27, 0.82)					
33	Kwak et al. (48)	Cross-sectional	Seoul, Republic of Korea	35.7 ± 0.32	Women			<30 nmol/l		Ultra-high performance	SBP≥ 140	2,098 premenopausal	1, 3, 5, 6, 7, 8, 11, 19, 22,23,24,25	9
		(NHANES 2007–2010)		62.3 ± 0.21	Premeno- pausal	238		30–49.99	1.00 (ref.)	liquid chromato graphy-tandem mass spectrometry	DBP ≥ 90 mm Hg, or the use of antihypertensive medication or physician diagnosis			
						586		≥ 50	1.23 (0·79, 1·92)			2,298 postmenopausal		
						1,274			0.64 (0·39, 1·02)					
					Postmenopausal			<30 nmol/l						
						195		30–49.99	1.00 (ref.)					
						505		≥ 50	0.68 (0·44, 1·04)					
						1,598			0.71 (0·47, 1·09)					
34	Lertratanakul et al. (49)	Cross-sectional	Toronto	39.3 ± 13.5	Both	873		Q1 (4–13 ng/ml)	1.00 (ref.)	NR	SBP≥ 140 DBP≥ 90 mm Hg, or taking current treatment for hypertension	Patients With Systemic Lupus Erythematous	1, 2, 3, 4, 11, 83	7
								Q2 (14–21)	0.83 (0.55, 1.25)					
								Q3 (22–30)	0.69 (0.44, 1.06)					
								Q4 (31–91)	0.49 (0.31, 0.77)					
35	Liu et al. (50)	Cross-sectional	China	65–112	Both	2,493				ELISA	BP≥ 130/85 mmHg or known treatment for hypertension	Elderly Chinese Individuals	1, 2, 3, 4, 5, 6, 7, 8, 12, 16	7
		Evidence from CLHLS				1,029		<20 ng/ml	1.00 (ref.)					
						890		20–30	1.23 (0.87, 1.75)					
						574		≥30	1.49 (0.97, 2.29)					
36	Margolis et al. (51)	Cohort (7 years of followup)	Minneapolis	50–79	Women	2,153	891	Q1 (<34.4 nmol/L)	1.00 (ref.)	CLIA	SBP≥ 140 DBP≥ 90 mm Hg, or self-report of medication prescribed for hypertension	Postmenopausal women	1, 3, 4, 5, 6, 7, 8, 11, 20, 37, 38, 84 85, 86	7
		Women's Health Initiative 1993–1998	USA					Q2 (34.4– <47.7)	0.91 (0.62, 1.32)					
								Q3 (47.7–64.7)	0.66 (0.46, 0.96)					
								Q4 (≥ 64.7)	0.86 (0.60, 1.23)					
37	Peng et al. (52)	Cross-sectional	Tangshan City	49.9 ± 12.5	Both	3,788				ELISA	SBP≥ 140 or	Coal mine workers	1, 4, 5, 6 , 7, 26, 58, 87, 88, 89	7
		(Kailuan cohort study)	China				717	<25nmol/L	1.39 (0.97, 1.99)		DBP≥ 90 mmHg on at least 3 different visits to the hospital taking antihypertensive agents			
							296	25–50	1.44 (0.99, 2.11)					
							54	≥50	1.00 (ref.)					
														
														
38	Reis et al. (53)	Cross-sectional	California, San Diego	44–96	Both	1,070				(CBP) recognition and	SBP≥ 130, DBP≥ 85 mmHg, or use of antihypertensive medication	Community-dwelling older adults	1, 5, 6, 7, 11, 16, 90	8
		(Rancho Bernardo Study cohort)			Men	410		Q1 (<87.5 nmol/l)	1.00 (ref.)	CLIA				
								Q2 (87.5– 97.4)	0.92 (0.41, 2.07)					
								Q3 (97.5–110.0)	0.90 (0.41, 1.96)					
								Q4 (110.1–126.2)	0.88 (0.42, 1.84)					
								Q5 ( ≥126.3)	1.28 (0.58, 2.81)					
														
					Women	660		Q1 (<77.5)	1.00 (ref.)					
								Q2 (77.5–92.4)	0.77 (0.40, 1.46)					
								Q3 (92.5– 103.7)	0.80 (0.44, 1.47)					
								Q4 (103.8–119.9)	0.81 (0.43, 1.53)					
								Q5 (≥ 120.0)	1.01 (0.53, 1.93)					
39	Shen et al. (54)	Cross-sectional	Henan, China	18–93	Both	1,539				ELISA	SBP≥ 140 DBP≥ 90 mmHg, or	Adults	1, 2, 5, 6, 7, 36	6
						68		<10 ng/ml	1.00 (ref.)		Use of antihypertensive medication			
						722		20–Oct	0.48 (0.28, 0.85)					
						387		20–30	0.37 (0.20, 0.66)					
						362		≥30	0.48 (0.27, 0.87)					
40	Snijder et al. (17)	Cross-sectional	Amsterdam	55–85	Both	1,205				CPBA	SBP> 140 and/or DBP> 90 mmHg and/or taking antihypertensive medication	Older men and women	1, 2, 5, 6, 7, 10, 11, 17	8
		LASA)				126		Q1 (<25.0 nmol/l)	0.89 (0.47, 1.69)					
						442		Q2 (25–50)	0.79 (0.50, 1.25)					
						410		Q3 (50–75)	0.75 (0.49, 1.15)					
						227		Q4 (>75)	1.00 (ref.)					
41	Song et al. (55)	Cross-sectional	Republic of Korea	58.0 ± 7.0	Women	778				CLIA	SBP≥ 130 or DBP≥ 85 mmHg, or taking medication	apparently healthy	1, 6, 7, 11	8
						193		Q1 (4.2–9.7 ng/ml)	1.81 (1.15, 2.85)			Post-menopausal women		
						199		Q2 (9.8–14.1)	1.91 (1.24, 2.94)					
						192		Q3 (14.2–19.8)	1.55 (1.02, 2.37)					
						194		Q4 (19.9–55.9)	1.00 (ref.)					
42	Steinvil et al. (56)	Cross-sectional	Israel	≥18	Both	34,874				RIA	ICD nine criteria	Adults	1	7
		(Health care maintenance organization 2001–2008)			Men	1,662		<15 ng/ml	1.11 (0.95, 1.30)					
						4,672		15–30	1.06 (0.93, 1.20)					
						1,841		>30	1.00 (ref.)					
														
					Women	5,816		<15 ng/ml	1.19 (1.09, 1.31)					
						15,341		15–30	1.07 (0.99, 1.15)					
						5,542		>30	1.00 (ref.)					
43	van Ballegooijen et al. (57)	Cohort	Amsterdam, The Netherlands	28–75	Both	5,066			HR	Liquid chromato- graphy-tandem mass spectrometry	SBP≥ 140 DBP≥ 90 mmHg or taking medication	Adults	1, 2, 4, 5, 6, 8, 11, 36, 45, 92	9
		/median follow-up of 6.4 years (2.3–9.0)				1,264		Q1 (6.7–40.7 nmol/l)	1.16 (0.95, 1.41)					
						1,270		Q2 (40.8–56.7)	1.07 (0.89, 1.27)					
						1,266		Q3 (56.8–73.7)	0.91 (0.75, 1.08)					
						1,266		Q4 (73.7–181.3)	1.00 (ref.)					
44	van Ballegooijen et al. (58)	Community–based prospective Cohort (MESA)/ 9 years follow-up	USA	45–84	Both	3,002				liquid chromato- graphy-mass spectroscopy	SBP≥ 140, DBP≥ 90 mmHg, or taking antihypertensive medication	Adults	1, 2, 3, 4, 5, 7, 8, 9, 45, 55, 93, 94, 95, 96, 97	8
						922		<20 ng/ml	1.13 (0.96, 1.33)					
						1,028		20–30	1.14 (0.98, 1.31)					
						1,052		>30	1.00 (ref.)					
45	Wang et al. (59)	Prospective Cohort/ 15.3–year follow-up	Boston	NR	Men	660			HR	Radio- immunosorbant assay	SBP≥ 140,	Adults	1 ,3 , 4, 5, 6, 7, 11, 21, 38, 39	6
			US			136		<50 nmol/l	1.00 (ref.)		DBP≥ 90 mmHg, or use of anti-hypertensive medication			
						244		50– <75	1.03 (0.75, 1.42)					
						178		75– <100	0.79 (0.56, 1.11)					
						102		100 ≤	0.94 (0.62, 1.40)					
46	Martins et al. (60)	Cross-sectional	United States	20 ≤	Both	15,088		<21 ng/ml	1.30 (1.13, 1.49)	RIA	SBP≥ 140,	Adults	1, 2, 3	8
		(based on NHANES III)						NR	NR		DBP≥ 90 mmHg			
								NR	NR					
								≥37	1.00 (ref.)					
47	Khader et al. (61)	Cross-sectional	Jordan	19 −90	Both	3,234				RIA	SBP≥ 130 or DBP≥ 85 mm Hg, or treatment of previously diagnosed hypertension	Adults	1, 2, 4, 7, 8, 10, 12, 18, 44, 46, 49	8
					Men	776		<30 ng/ml	1.008 (0.83, 1.22)					
					Women	2,458		≥30	1.00 (ref.)					
48	Jeenduang et al. (62)	Cross-sectional	Southern	62.6 ± 9.76	Women	340				ECLIA	SBP≥ 130	Postmenopausal women	1, 4, 5, 6, 7, 8, 13, 20, 21, 27,35	8
			Thailand			194		<30 ng/ml	1.092 (0.663, 1.798)		and/or DBP			
						146		30 ≤	1.00 (ref.)		≥ 85 mmHg			
49	Gupta et al. (63)	Cross-sectional	USA	20 ≤	Both	461		≤ 45.4 nmol/l	1.21 (0.76, 1.92)	RIA	Pre-HTN (resting SBP: 120–139 mm Hg and/or DBP:	Healthy adult	1, 2 ,4	8
		(based on NHANES 2001–2006)				160		>45.4	1.00 (ref.)		80–89 mm Hg)			
														
														
50	Gupta et al. (63)	Cross-sectional	LA	20 ≤	Both	591		≤ 60.4 nmol/l	1.20 (0.60, 2.39)	RIA	Pre-HTN	Healthy Mexican Americans	1, 2,4	8
		(based on NHANES 2001–2006)	USA			197		>60.4	1.00 (ref.)		(resting			
											SBP: 120–139 mm Hg and/or DBP: 80–89 mm Hg)			
51	Gupta et al. (64)	Cross-sectional	LA	20 ≤	Both	1,272		≤ 76.3 nmol/l	1.61 (1.23, 2.10)	NR	Pre-HTN (SBP: 120–139 mmHg and/or DBP: 80–89 mmHg)	Healthy disease-free Caucasians	1, 2,4	6
		(based on NHANES 2001–2006)	USA			439		>76.3	1.00 (ref.)					
52	Esteghamati et al. (65)	Cross-sectional	Tehran	>18	Both	4,391		<20 ng/ml	1.34 (0.74, 2.41)	RIA	SBP≥ 130 or DBP≥ 85 mmHg, or a history of antihypertensive-drug use	Metabolically healthy obesity	1, 2,4	9
			Iran					≥20	1.00 (ref.)					
												Metabolically unhealthy obesity		
														
									1.66 (1.50, 1.82)					
									1.00 (ref.)					
53	Contreras–Manzano et al. (66)	Cross-sectional	Mexico	20– 49	women	3,260		<50 nmol/l	1.24 (0.84– 1.81)	Chemilumi nescence microparticle immunoassay	SBP> 140 and/or a DBP> 90 mmHg or previous diagnosis by a physician of hypertension	Mexican Women of Reproductive Age	3, 10, 58, 100, 101, 102, 103,104, 105, 106, 107, 108, 109, 110	7
		National Health and Nutrition Survey (ENSANUT 2012)						≥50	1.00 (ref.)					
54	Caro et al. (67)	Cross-sectional	San Juan, Puerto Rico	21–51	Both	219		<30 ng/ml	1.11 (0.35, 3.51)	EIA	HTN (SBP≥ 140 or DBP ≥ 90mmHg or antihypertensive medication)	Adults	1, 2, 4, 20,111	8
				41.5 ± 13.9				≥30 ng/ml	1.00 (ref.)		Pre-HTN (SBP 120–139, DBP 80–89)			
														
														
														
								<30 ng/ml	0.88 (0.49, 1.60)					
								≥30 ng/ml	1.00 (ref.)					
														
55	Zhao et al. et al. (68)	Cross-sectional	Atlanta	20 ≤	Both	7,228			(PR)	RIA	SBP≥ 140 or DBP≥ 90mmHg	Non-institutionalized civilian United States population	1, 2, 3, 4, 6, 7, 8, 9, 12, 18, 40, 43, 45, 46, 47, 48, 53, 94, 97, 112,113	9
		(based on NHANES)2003–2006	USA			1,665		<15 ng/ml	1.00 (ref.)		or a history of antihypertensive-drug use			
						1,420		15– <20	0.89 (0.80, 0.99)					
						1,536		20– <25	0.89 (0.79, 1.00)					
						1,250		25– <30	0.89 (0.81, 0.98)		Pre-HTN (SBP 120–139, DBP 80–89)			
						1,357		≥30	0.82 (0.73, 0.91)					
														
						1,665		<15 ng/ml	1.00 (ref.)					
						1,420		15– <20	0.83 (0.72, 0.96)					
						1,536		20– <25	0.88 (0.74, 1.07)					
						1,250		25– <30	0.87 (0.73, 1.03)					
						1,357		≥30	0.80 (0.69, 0.92)					
56	Sumriddetchka- jorn et al. (69)	Cross-sectional	Bangkok, Thailand	35–54	Both	274	137	≤ 28ng/ml	0.56 (0.33, 0.96)	NR	SBP≥ 140 and/or	137 Hypertensive cases and 137 normotensive controls	1, 2, 4, 54, 56	4
								>28	1.00 (ref.)		DBP ≥ 90mmHg			
57	Shin et al. (70)	Cross-sectional	Seoul, Korea	50– 79	Women	4,107		<15 (ng/ml)	1.28 (1.02, 1.61)	RIA	SBP ≥ 140, or DBP ≥ 90mmHg or taking antihypertensive medication	Postmenopausal .women	1, 4, 5, 6, 7, 8, 9, 11, 15, 17, 45	9
		(based on KNHANES V 2010–2012)						≥15	1.00 (ref.)					
58	Pannu et al. (71)	Cross-sectional	Melbourne	18–75	Both	3,387		Per 10 nmol/l increase	1.02 (0.97, 1.07)	Automated direct competitive chemiluminescent immunoassay	SBP≥ 130	Adults	1, 2, 3, 4, 5, 6, 7, 8, 9, 11, 16, 19, 25, 28, 29	9
		based on Victorian Health Monitor (VHM) survey	Victoria											
			Australia						0.97 (0.92, 1.01)		DBP≥ 85			
											mmHg			
											or on antihypertensive medications			
														
														
59	Li et al. (72)	Cross-sectional	Yunnan Province, China	20–83	Both	1,206		Per 1 ng/ml increase		RIA	SBP ≥140 or DBP ≥ 90mmHg, taking antihypertensive medication	Participants without antihypertensive treatment	1, 2, 4, 5, 6, 36, 45	7
					Men	728		in baseline	1.00 (0.98, 1.02)					
														
					Women	478			1.00 (0.97, 1.04)					
60	Kwak et al. (48)	Cross-sectional	Republic of Korea	≥20	Both	2,591		Per 1 ng/ml increase in baseline	0.97 (0.94, 0.99)	RIA	SBP ≥140	Adults	1, 2, 4, 5, 6, 7, 8, 77	9
		(KNHANES) 2011–2012									DBP ≥ 90mmHg or on antihypertensive medication			
61	Gagnon	Cohort	Australia	≥25	Both	4,164		Per 10–ng/ml decrease in baseline	1.03 (0.94, 1.14)	CLIA	SBP≥ 130	Adults	1, 2, 3, 5, 7, 8, 11 16, 17, 38, 45, 83	8
	2012	5 years of follow-up:									DBP≥ 85			
		(The Australian Diabetes, Obesity and Lifestyle study,									mmHg			
		AusDiab)									or on antihypertensive medications			
62	Dong et al. (73)	Cross-sectional	China	≥18	Both	837		Per 10 nmol/l increase in baseline	0.66 (0.38, 1.16)	ELISA	SBP≥ 130	Peritoneal dialysis patients	1, 2, 20, 43, 46, 50, 51, 114, 115, 116, 117	6
											DBP≥ 85			
											mmHg (or drug treatment)			
63	Chen et al. (74)	Cross-sectional	Beijing, China	60_102	Both	1,245		Per 1 ng/ml increase in baseline	0.98 (0.97, 0.99)	Chemilumi- nescence assay	SBP ≥140	Elderly Chinese Population	1, 2, 4, 5, 6, 18, 43, 45, 46, 58, 89, 115, 118	9
											DBP ≥ 90mmHg or a taking antihypertensive drug			
64	Chen et al. (75)	Cross-sectional	China	54.9	Both	10,655		Per 10 nmol/l increase in baseline	1.043 (1.004, 1.084)	CLIA	SBP≥ 130	Adults	1, 2, 5, 9, 15, 17, 54, 56, 58	8
											DBP≥ 85			
											mmHg (or drug treatment)			
65	Skaaby et al. (76)	Cohort	Denmark	29.7–61.2	Both	2,571	403	per 10 nmol/l increase in baseline	1.01 (0.97, 1.05)	HPLC	SBP> 140, DBP> 90 mm Hg or treatment of previously diagnosed	Adults	1, 2, 4, 5, 6, 7, 8, 11, 17, 18, 97, ,119, 120, 121	9
		5 years of followup									hypertension			
														
66	Sabanayagam et al. (77)	Cross-sectional	USA	>20	Both					RIA	Prehypertension: SBP 120–139 mm Hg or DBP 80–89 mm Hg	Adults	1, 3, 4, 5, 6, 7, 38, 43, 45, 122	9
		(NHANES III)			Men	800		Q1 ( ≤ 17.7 ng/ml)	1.53 (1.13, 2.07)					
						1,040		Q2 (17.8– 24.6)	1.28 (0.98, 1.66)					
						1,207		Q3 (24.7– 32.4)	1.07 (0.80, 1.44)					
						1,242		Q4 (>32.4)	1.00 (ref.)					
														
					Women	1,482		Q1 ( ≤ 17.7 ng/ml)	1.44 (1.03, 2.00)					
						1,278		Q2 (17.8– 24.6)	1.23 (0.93, 1.62)					
						1,099		Q3 (24.7– 32.4)	1.19 (0.89, 1.61)					
						1,067		Q4 (>32.4)	1.00 (ref.)					
67	Vacek et al. (78)	Cross-sectional	Kansas	58.3 ± 14.9	Both	10,899		<30 ng/ml	1.40 (1.285, 1.536)	CLIA	SBP> 140, DBP> 90 mm Hg	Adults	NR	8
			USA					≥30	1.00 (ref.)					
														
68	Mateus-Hamdan et al. (79)	Cross-sectional	France	85.87 ± 5.90	Both	284		Per 1 nmol/l increase in baseline	1.01 (0.99, 1.03)	RIA	SBP> 140, DBP> 90 mm Hg	Elderly inpatients	1, 2, 11, 20, 22, 50, 77, 126, 127, 128, 129, 130	7
69	Ford et al. (16)	Cross-sectional	Boston	20 ≤	Both	8,421		≤ 48.4 nmol/l	1.00 (ref.)	RIA	SBP≥ 130	Adults	1, 2, 3, 5, 6, 8, 11, 16, 21, 31, 40, 43, 53,	7
		(based on NHANES III, 1988–1994)						48.5– 63.4	1.17 ( 0.95, 1.44)		DBP≥ 85			
								63.5– 78.1	1.00 (0.77, 1.30)		mmHg			
								78.2– 96.3	1.16 (0.85, 1.59)					
								≥96.4	1.07 (0.77, 1.50)					
70	Muldowney et al. (80)	Cross-sectional	Multi Country	20–40	Both	195		T1 ( ≤ 42.5nmol/l)	0.87 (0.35, 2.20)	ELISA	SBP≥ 130 mmHg	Participants in a weight loss dietary intervention study	1, 2, 5, 10, 16, 17, 18, 20, 22, 111	7
			Iceland (64°N;), Ireland (51°N;) and Spain (42°N;)					T2 (42.51–63.0)	NR					
								T3 (>63.0)	1.00 (ref.)					
									1.21 (0.16, 8.87)					
									NR		DBP≥ 85			
									1.00 (ref.)		mmHg			
71	Piantanida et al. (81)	Cross-sectional	Italy	51 ± 13	Both	196		<10 ng/ml	1.65 (0.7, 4.0)	CLIA	SBP≥ 130	Caucasian obese adults	4	6
								20–Oct	3.2 (1.5, 7.0)		DBP≥ 85			
								≥20	1.00 (ref.)		mmHg			

a *Adjustments: 1, Age or age range; 2, Gender; 3, Race or ethnicity/ country of birth; 4, BMI or BMI category; 5, Smoking (status); 6, Alcohol; 7,Physical activity or exercise; 8, Education; 9,Income; 10, Region; 11,Time of blood drown(season/month/week); 12, Marital status; 13, Religion; 14, Occupation; 15, Residency/ residential district; 16, The rest of the individual components of the MetS; 17, Waist Circumference/ abdominal obesity; 18, Parathyroid hormone; 19, Total energy intake; 20, Total vitamin D intake/ supplementation; 21, Multivitamin supplementation; 22, Calcium intakes/ supplementation; 23, Potassium intakes; 24, Sodium intakes; 25, Magnesium intakes;26, Salt intake (low, medium, high); 27, Fish oil intake; 28, Zinc intake; 29, Fiber intake; 30, Eggs intake;31, Consumption of fruits, vegetables, dairy, red meat, whole grains and refined sugar; 32, Sum of total fruit and vegetable Healthy Eating Index scores; 33, Diet quality score; 34, Sleeping pattern; 35, The use of sunscreen; 36, Family history of hypertension; 37, (family) History of cardio-metabolic diseases; 38, History of diabetes; 39, History of hypercholesterolemia; 40, Serum cotinine; 41, Baseline cardio-metabolic diseases; 42, Year of blood draw ;43, Serum C-reactive protein; 44, Serum creatinine; 45, eGFR; 46, Serum calcium; 47, Serum sodium; 48, Serum potassium; 49, Serum magnesium levels; 50, Serum albumin; 51, Serum phosphorus; 52, Serum iron; 53, Serum total cholesterol; 54, HDL-C; 55, LDL-C; 56, Triglycerides; 57, HbA1c ranges; 58, Diabetes Mellitus; 59, HT; 60, Time of menopause; 61, Waist to Hip Ratio; 62, Work related physical activity; 63, Recruitment location; 64, Ultraviolet radiation index at participants recruitment location; 65, RI (defined as eGFR <60 mL/min/1.73 m2); 66, 1,25 (OH)2D; 67, Study center; 68, Different casecontrol study vitamin D analysis; 69, Day of menstrual cycle if premenopausal; 70, Hour of the blood collection; 71, Oral contraceptive use; 72, Serum uric acid; 73, Menopausal status; 74,25(OH)D/IGF-1 as relevant; 75, Birth; 76, Adult social class; 77, Antihypertensive medication; 78, Laboratory of 25(OH)D analysis; 79, Number of years smoked; 80, Number of cigarettes smoked per day; 81, The presence of diseases; 82, Length of residence in South Korea; 83, Country (Korea, UK, US, or other); 84, Clinical center; 85, Calcium/vitamin D trial assignment; 86, Blood pressure at enrollment; 87, Work type (mental work, physical work); 88, Work environment (surface or underground); 89, Hyperlipidemia; 90, In women, hormone therapy; 91, %Fat mass; 92, 24-h urinary albumin excretion; 93, Clinic site; 94, Diabetes status; 95, Non-steroidal anti-inflammatory drug use; 96, Cyclooxygenase-2 inhibitor use; 97, Albumin creatinine ratio; 98, Diastolic blood pressure; 99, Low HDL-cholesterol; 100, Area (urban/rurality); 101, Wellbeing index tertiles; 102, HDL-C (<50 mg/dL); 103, TG (<150 mg/dL); 104, TC (<200 mg/dL); 105, CRP (<5 mg/dL); 106, Hcy (<10 umol/L); 107, Sedentarism; 108, Overweightobesity; 109, insulin resistance; 110, Acute myocardial infarction; 111, Sun exposure; 112, Coronary heart disease; 113, Dietary supplement use; 114, Dialysis duration; 115, Serum hemoglobin; 116, Total Kt/V urea; 117, Residual renal function; 118, Fasting glucose; 119, Randomization status; 120, Diet; 121, 5-year changes in BMI; 122, Total to high-density lipoprotein cholesterol ratio; 123, Ratio of non-HDL to HDL cholesterol; 124, Stroke in parents; 125, Atrial fibrillation; 126, The number of chronic diseases (i.e. diseases lasting at least 3 months or running a course with minimal change); 127, Drugs taken per day; 128, Corticosteroid drugs; 129, TSH; mUI/L; 130, Creatinine clearance (mL/min); 131, Peripheral vascular disease*.

*CLIA, Chemiluminescent immunoassay; RIA, Radioimmunoassay; ELISA, Enzyme-linked immunosorbent assay; ECLIA, electrochemiluminescence immunoassay; EIA, Enzyme immunoassay; CPBA, Competitive protein binding ; DEIA, Direct enzyme immunoassay; HPLC, High-performance liquid chromatography; SBP, Systolic blood pressure; DBP, Diastolic blood pressure; HTN, Hypertension, SLE, Systematic lupus erithematous*.

### Meta-Analysis of Highest vs. Lowest Vitamin D Level in Relation to Hypertension in Prospective Studies

**Figure 2:**
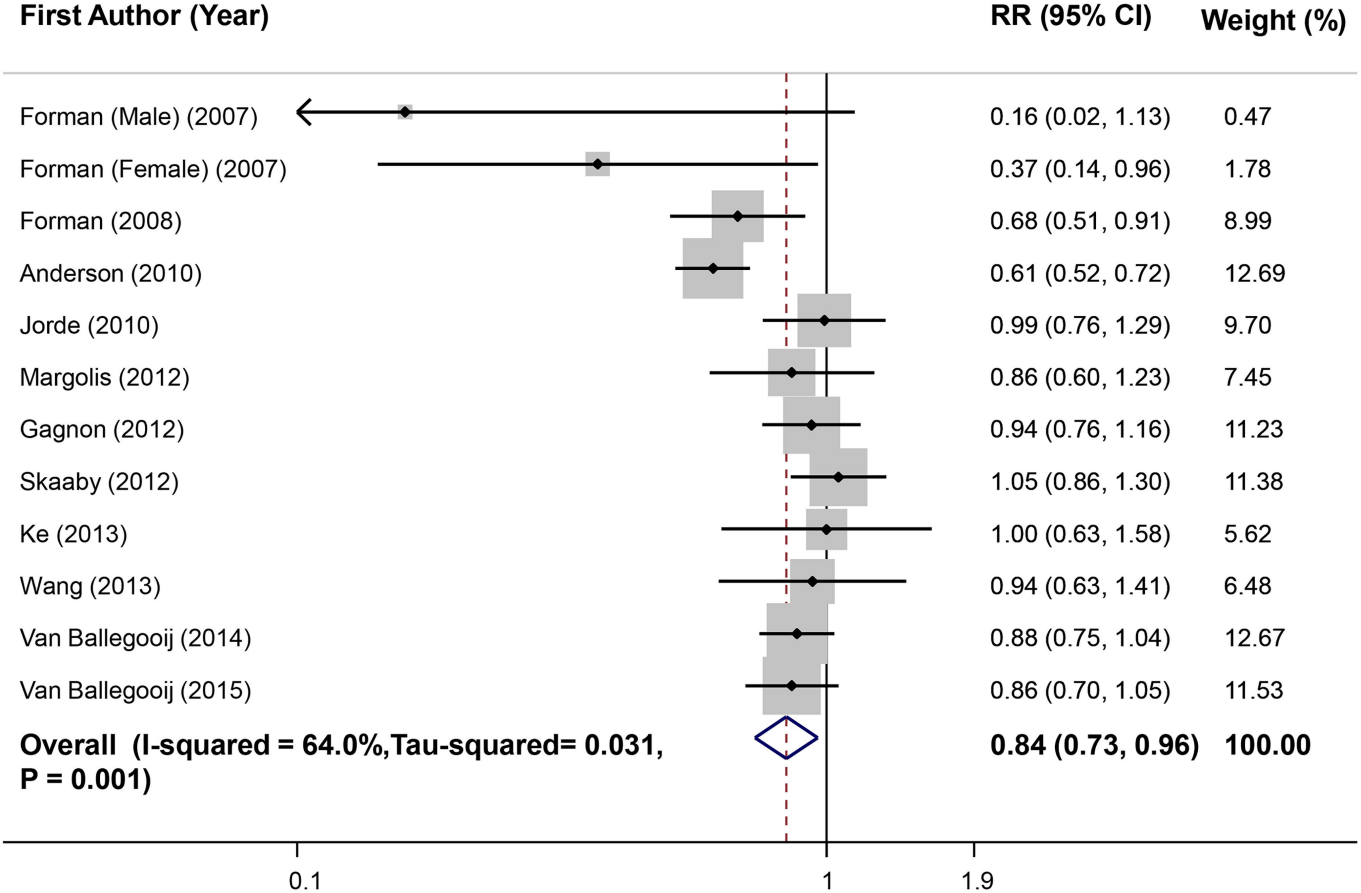
Forest plot of prospective studies that examined the association between highest vs. lowest level of serum vitamin D and risk of hypertension (HTN).

Combination of 12 effect sizes from 11 studies (*n* = 66,757) led to an overall effect of 0.84 (0.73, 0.96) that showed a 16% decrease in risk of hypertension in participants who had a high level of serum vitamin D compared with those with low level (Figure 2). Heterogeneity was moderate (*I*^2^ = 64%, Tau^2^ = 0.031, *P* = 0.001). Subgroup analyses were conducted to find the source of heterogeneity and the findings are reported in Table 2. None of the covariates could completely explain the observed heterogeneity. Then, excluding one study of Anderson et al. (39) removed the observed heterogeneity (*I*^2^ = 24.3%, *P* = 0.21), without significant change in the overall estimate (RR = 0.89; 95%CI: 0.81, 0.99). Sensitivity analysis showed that excluding each investigation had no significant effect on pooled RR. Also, there was no asymmetry in the funnel plot and no evidence for publication bias (Begg's test = 0.49, Egger's test = 0.64) (Supplementary Figure 1A).

**Table 2:** Results of subgroup-analysis for circulating vitamin D levels and risk of hypertension in Cohort studies.

	**No. of effect sizes**	**RR (95% CI)**	**I^**2**^ (%)**	***P* within^a^**	***P* between^b^**
**Overall**	12	0.84 (0.73, 0.96)	64	0.001	
**Gender**					0.332
Male	3	0.89 (0.58, 1.37)	37.7	0.201	
Female	3	0.71 (0.53, 0.95)	32.1	0.229	
Both	6	0.87 (0.73, 1.03)	77.5	<0.001	
**Comparison vitamin D levels**					<0.001
Q5 vs. Q1	1	1.00 (0.63, 1.58)	–	–	
Q4 vs. Q1	4	0.90 (0.79, 1.04)	00.0	0.853	
T3 vs. T1	5	0.90 (0.74, 1.09)	52.9	0.075	
SUF vs. DIF	2	0.63 (0.54, 0.72)	00.0	0.526	
**Time of Blood draw adjustment**					0.016
Adjusted	7	0.90 (0.82, 1.00)	5.1	0.388	
Not adjusted	5	0.73 (0.54, 0.98)	78.3	0.001	
**Age, gender, BMI adjustment**					0.007
Adjusted	6	0.90 (0.77, 1.06)	45.5	0.103	
Not adjusted	6	0.80 (0.66, 0.97)	64.4	0.015	
**Representative**					0.861
Representative	8	0.84 (0.70, 1.00)	74.8	<0.001	
Not representative	4	0.82 (0.68, 0.98)	00.0	0.427	
**Quality status^c^**					0.002
High quality	9	0.88 (0.78, 0.99)	38.1	0.115	
Low quality	3	0.79 (0.55, 1.13)	70.8	0.001	

a *P for heterogeneity, within subgroup*.

b *P for heterogeneity, between subgroups.*

c *Quality scores were according to: Newcastle-Ottawa Scale*.

### Dose–Response Meta-Analysis of Serum Vitamin D and Risk of Hypertension in Prospective Studies

**Figure 3:**
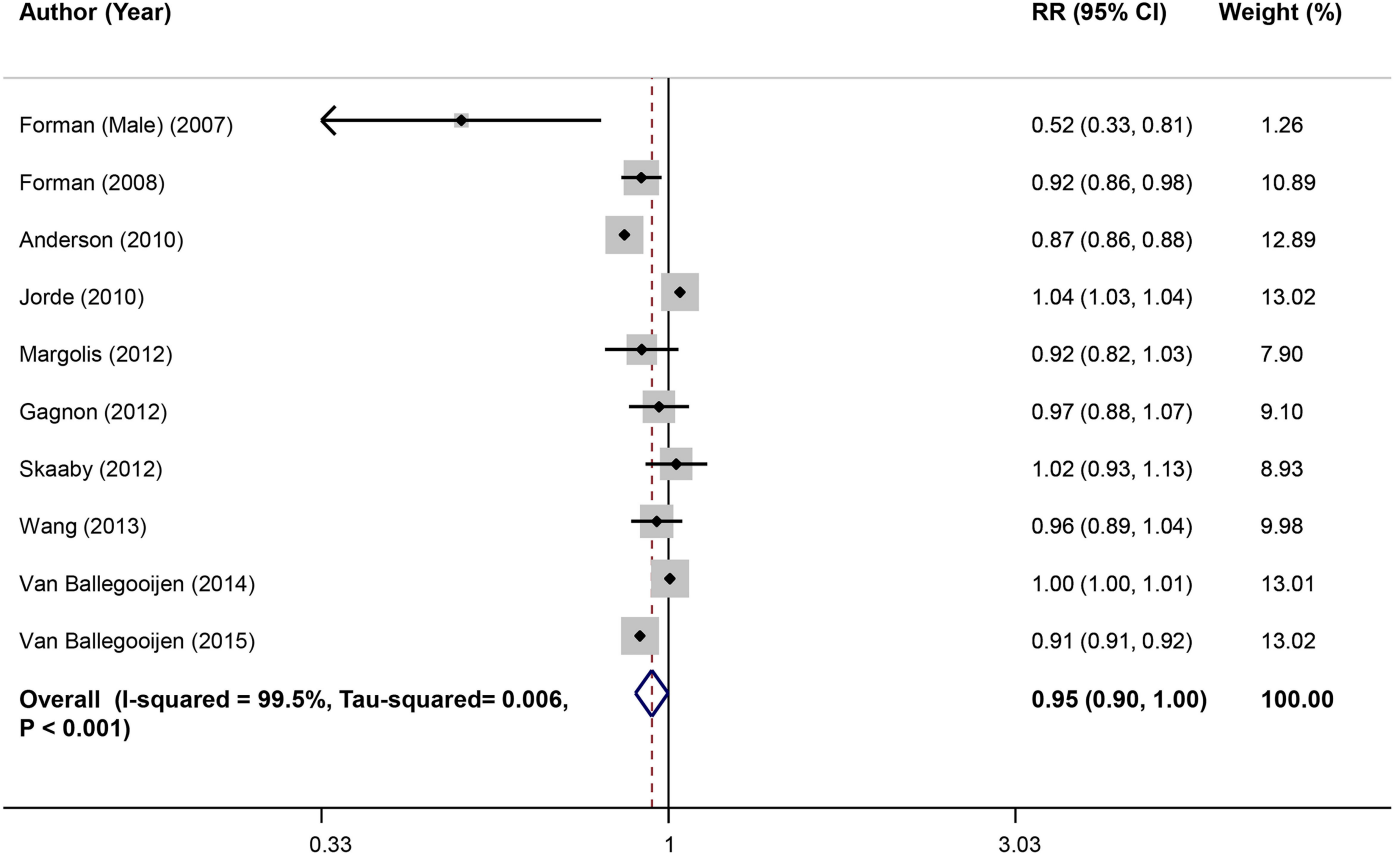
Linear dose–response meta-analysis of serum vitamin D and risk of HTN in prospective studies.

**Figure 4:**
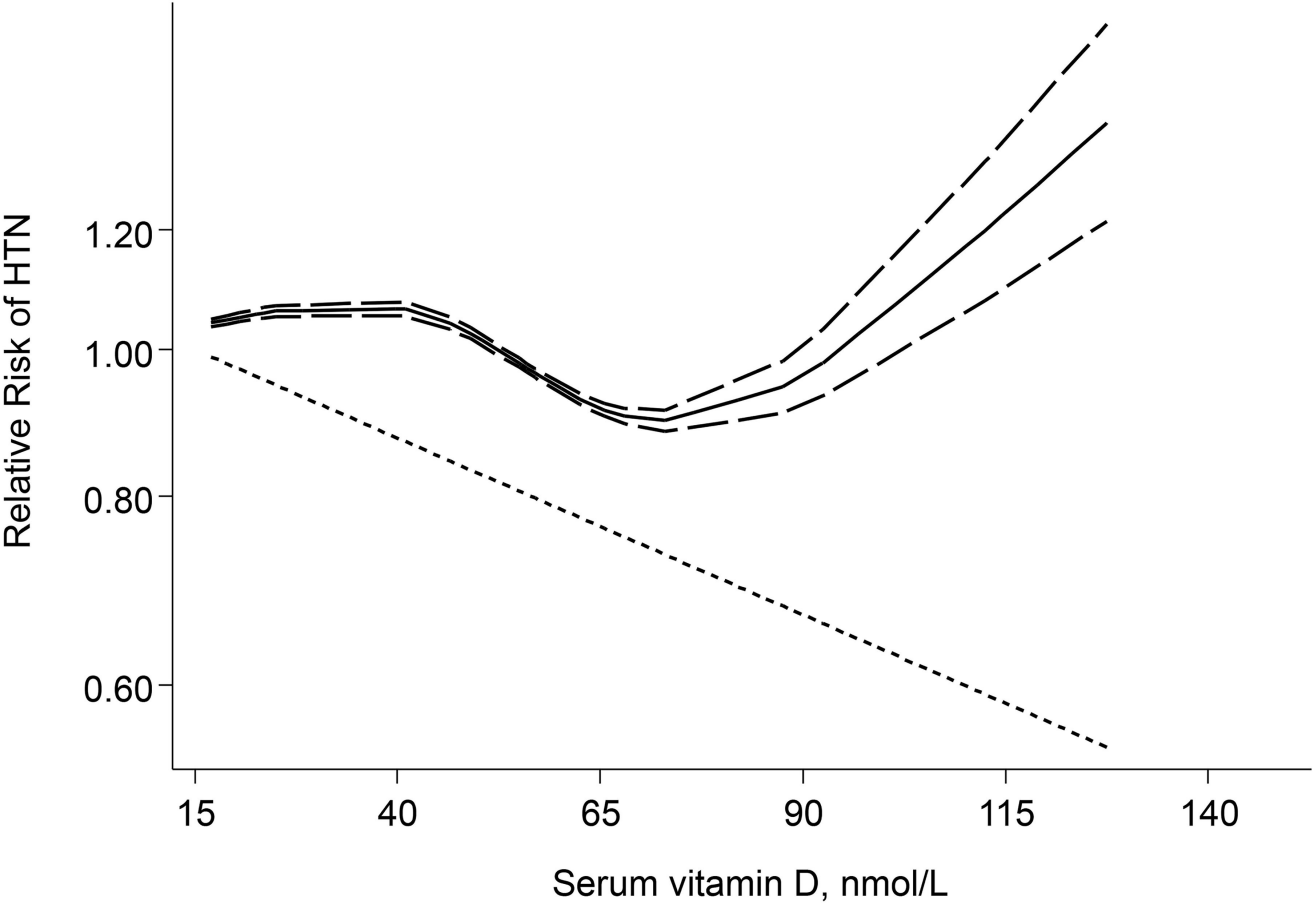
Non-Linear dose–response meta-analysis of serum vitamin D and risk of HTN in prospective studies.

Combining effect sizes of 10 studies involving a total of 63,602 individuals and 25,019 cases of hypertension showed that each 25 nmol/L increase in serum vitamin D level resulted in a 5% reduction in risk of hypertension (RR: 0.95; 95% CI: 0.90, 1.00) (Figure 3). Also, a significant non-linear association between serum vitamin D levels and hypertension was observed (*P*_non−linearity_ <0.001). A reduction trend in risk of hypertension was observed for serum vitamin D levels between 45 and 70 nmol/L, although for higher vitamin D levels the risk did not decrease anymore and eventually started increasing (Figure 4).

### Meta-Analysis of Highest vs. Lowest Vitamin D Level in Relation to Hypertension in Cross-Sectional Studies

**Figure 5:**
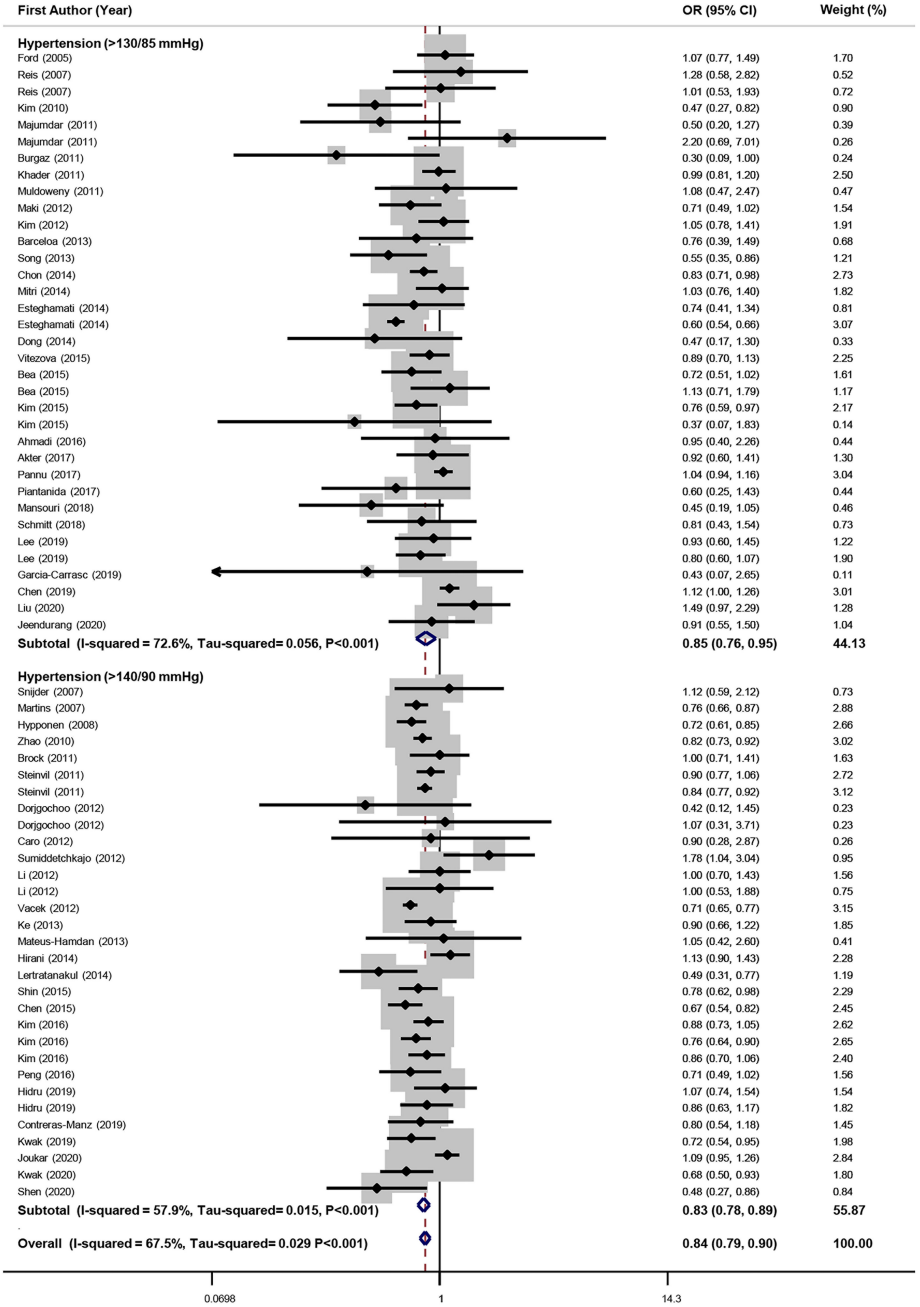
Forest plot of cross-sectional studies that examined the association between highest vs. lowest level of serum vitamin D and odds of HTN.

The association between serum vitamin D and odds of hypertension was examined in 56 investigations; 66 effect sizes were included in this analysis (*n* = 248,657). Meta-analysis determined that highest level of vitamin D in comparison to the lowest level was associated with a 16% significant decrease in odds of hypertension in cross-sectional studies (95%CI: 0.79, 0.90). Heterogeneity was significant (*I*^2^ = 67.5%, Tau^2^ = 0.029, *P* < 0.001). Subgroup analyses were conducted according to different confounders and the findings are presented in Table 3. The definition of hypertension (BP ≥ 140/90 vs. ≥ 130/85 mmHg) had no effect on results (for BP ≥ 140/90: OR = 0.83; 95%CI: 0.78, 0.89 and for BP ≥ 135/80 OR = 0.85; 95%CI: 0.76, 0.95) (Figure 5). Also, in both representative and not representative studies, a significant reduction in the odds of hypertension was observed (for representative studies: OR = 0.86; 95%CI: 0.80, 0.92 and for not representative studies: OR = 0.78; 95%CI: 0.69, 0.88). Although in most of the subgroups significant associations between vitamin D level and hypertension were found, none of the confounders could fully explain the observed heterogeneity. Sensitivity analysis determined that the exclusion of each study did not significantly affect the overall estimate. No significant publication bias was observed (Begg's test = 0.44, Egger's test = 0.84) (Supplementary Figure 1B).

**Table 3:** Results of subgroup-analysis for circulating vitamin D levels and odds of hypertension in cross-sectional studies.

	**No. of effect sizes**	**OR (95% CI)**	**I^**2**^ (%)**	***P* within^a^**	***P* between^b^**
**Overall**	66	0.84 (0.79, 0.90)	67.5	<0.001	
**Asian vs. Non-Asian**					0.716
Asian	39	0.84 (0.77, 0.91)	72.2	<0.001	
Non-Asian	27	0.85 (0.78, 0.94)	58.7	<0.001	
**Development status**					0.337
Developed	41	0.83 (0.79, 0.88)	49.7	<0.001	
Developing	25	0.87 (0.74, 1.01)	79.9	<0.001	
**Gender**					0.072
Male	12	0.92 (0.82, 1.02)	17.8	0.269	
Female	17	0.82 (0.77, 0.87)	00.0	0.735	
Both	37	0.84 (0.76, 0.92)	78.7	<0.001	
**Comparison Vitamin D levels**					<0.001
Q5 vs. Q1	9	0.90 (0.76, 1.07)	42.1	0.087	
Q4 vs. Q1	16	0.79 (0.73, 0.86)	26.6	0.156	
T3 vs. T1	26	0.89 (0.82, 0.97)	59.2	<0.001	
SUF vs. DIF	15	0.82 (0.69, 0.96)	79.8	<0.001	
**Outcome definition**					0.034
Blood Pressure ≥ 140/90	31	0.83 (0.78, 0.89)	57.9	<0.001	
Blood Pressure ≥ 130/85	35	0.85 (0.76, 0.95)	72.6	<0.001	
**Health status**					<0.001
Healthy	54	0.86 (0.81, 0.91)	62.9	<0.001	
Unhealthy	12	0.74 (0.61, 0.90)	44.4	0.048	
**Time of Blood draw adjustment**					0.003
Adjusted	35	0.85 (0.79, 0.91)	50.6	<0.001	
Not adjusted	31	0.85 (0.77, 0.93)	75.5	<0.001	
**Age, gender, BMI adjustment**					0.003
Adjusted	30	0.86 (0.77, 0.95)	76.5	<0.001	
Not adjusted	36	0.84 (0.78, 0.90)	54.3	<0.001	
**Representative**					0.136
Representative	45	0.86 (0.80, 0.92)	74.7	<0.001	
Not Representative	21	0.78 (0.69, 0.88)	14.9	0.265	
**Quality Status^c^**					0.030
High quality	36	0.82 (0.76, 0.89)	76.4	<0.001	
Low quality	30	0.89 (0.81, 0.99)	38.3	0.018	

a *P for heterogeneity, within subgroup*.

b *P for heterogeneity, between subgroups*.

c *Quality scores were according to: Newcastle–Ottawa Scale*.

### Dose–Response Meta-Analysis of Serum Vitamin D and Risk of Hypertension in Cross-Sectional Studies

Combing effect sizes of 30 studies involving a total of 139,685 individuals and 40,178 cases of hypertension showed that each 25 nmol/L increase in serum vitamin D level resulted in a 6% reduction in risk of hypertension (OR = 0.94; 95% CI: 0.90, 0.99) (Supplementary Figure 2). Also, a significant non-linear association between serum vitamin D levels and hypertension was seen (*P*_non−linearity_ <0.001); such that a reduction trend in odds of hypertension for serum vitamin D levels was observed between levels of 40–75 nmol/L, higher vitamin D levels did not reduce odds of HTN (Supplementary Figure 3). When the analysis was restricted to 21 cross-sectional studies with representative populations (133.497 general adult population with 37,341 cases of hypertension), we found that each 25 nmol/L increase in circulating vitamin D concentration reduced the risk of HTN by 3% (RR: 0.97; 95%CI: 0.95, 0.99) (Supplementary Figure 4). Also, a significant non-linear association between serum vitamin D levels and hypertension was observed (P_non−linearity_ <0.001). As shown in Supplementary Figure 5, a U-shaped relationship was found.

### Meta-Analysis of Highest vs. Lowest Vitamin D Level in Relation to Pre-hypertension in Cross-Sectional Studies

Combining 9 effect sizes from 7 studies (*n* = 21,242) revealed that the highest level of vitamin D was associated with a 25% significant reduction in odds of pre-hypertension, compared to the lowest level (0.95%CI: 0.68, 0.83), without heterogeneity between studies (*I*^2^ = 0.0%, Tau^2^ = 0.000, *P* = 0.44) (Supplementary Figure 6). Sensitivity analysis was carried out and excluding each investigation had no significant effect on the overall estimate. No evidence for publication bias was seen (Begg's test = 0.40, Egger's test = 0.82) (Supplementary Figure 1C).

### Quality of the Evidence

GRADE evidence profile for serum vitamin D concentration in relation to hypertension and pre-hypertension are presented in Supplementary Tables 5 and 6. The certainty of the evidence was rated as “high quality” for both cohort and cross-sectional studies for serum vitamin D concentration in relation to hypertension, and “moderate quality” for cross-sectional studies that investigated serum vitamin D concentration in relation to pre-hypertension. For serum vitamin D–hypertension relation, the endpoint for both cohort and cross-sectional studies was upgraded for domains of “risk of bias” and “inconsistency.” For cohort studies, 95% CI of overall effect contained a minimal value of 0.75; so, the certainty of the evidence was downgraded for “imprecision.” Both cohort and cross-sectional studies had also reported essential data for dose–response analysis; so, the endpoint of these investigations was upgraded for “other considerations.“ For pre-hypertension, the endpoint for cross-sectional studies was upgraded for the ”risk of bias “domain. Also, 95% CI of overall effect contained a minimal value of 0.75; so, the certainty of the evidence was downgraded for ”imprecision.“ Included studies in the analysis of prehypertension did not provide enough data for dose–response analysis and the endpoint for these studies was downgraded for “other considerations.”

## Discussion

In this meta-analysis, we found an inverse significant association between serum vitamin D concentrations and risk of hypertension in the adult population, when we compared the highest level of serum vitamin D vs. the lowest level, in both prospective cohort and cross-sectional studies. This inverse association was independent of hypertension definition (BP ≥ 140/90 vs. ≥ 130/85 mmHg). Also, dose–response analysis showed a significant linear and non-linear relationship between serum vitamin D and risk of hypertension.

Hypertension is one of the most prevalent chronic diseases all over the world and imposes a great economic burden on health care systems (88, 89). BP control rates are far from satisfactory worldwide, while hypertension is a main preventable cause of CVD and all-cause mortality (88). We demonstrated that normal levels of serum vitamin D concentrations were associated with a lower risk of hypertension, but the lowering risk did not continue after increasing serum vitamin D from normal levels. This finding could be particularly important for vitamin D-deficient adults to have a successful treatment, while they should avoid receiving extra vitamin D supplements.

In line with our meta-analysis, several previous systematic reviews and meta-analyses have evaluated the association between serum vitamin D levels and the risk of different non-communicable diseases. In 2013, a dose–response meta-analysis on 5 prospective cohorts revealed that each 10 ng/mL (or 25 nmol/L) increment in serum vitamin D levels was associated with a 12% decreased risk of future hypertension (90). However, almost all of the included studies were conducted in United States which made it impossible to generalize the finding to other populations and the number of included individuals (*n* = 6,716) and cases of HTN (*n* = 2,371) were limited (90). A recent dose–response analysis indicated that each 25 nmol/L increment in serum vitamin D concentration was related to 8% reduced risk of abdominal obesity (91). Furthermore, another dose–response analysis of prospective studies reported that a decrease of 10 nmol/L vitamin D was associated with a 7% increment in the risk of CVD mortality in older adults (92), although the small overall sample size of the study (21,079 participants) might have a certain impact on the estimated results in the mentioned analysis (92). Another meta-analysis on prospective cohorts showed that decreased vitamin D levels were associated with a 54% increment in risk of CVD mortality with no significant results among gender subgroups (93). Some studies revealed that the CVD mortality was higher in vitamin D-deficient men (94) and some others confirmed a lower mortality rate in vitamin D-deficient women than men (95). In the current meta-analysis, an inverse significant association was found in women in both cohort and cross-sectional studies, while no significant relation was observed in the male population. However, it should be considered that a few number of effect sizes were available from the male population and almost half of the included studies did not provide separate reports for men and women. Taken these findings together, future studies should provide gender-stratified analysis to shed a light on the gender-specific relations.

In a meta-analysis of randomized controlled trials, a small reduction in DBP was seen in response to vitamin D supplementation in hypertensive patients but had no significant effect on normotensive individuals (96). Another meta-analysis on 8 trials with 917 participants indicated that vitamin D supplementation had a moderate SBP-lowering effect (-1.964 mmHg) without significant effect of DBP. So, this study suggested that vitamin D supplementation could not be used as an antihypertensive agent. While interpreting the results of the mentioned meta-analysis, it should be taken into account that the included trials were performed on both hypertensive and normotensive individuals without considering their baseline 25(OH)D status. Also, the number of included studies was limited and ethnicity and latitude, as 2 effective factors on baseline vitamin D concentrations, had not been considered (97). Another meta-analysis of cohort studies and randomized controlled trials suggested a 7% decrease in risk of hypertension per 25 nmol/L increment in serum vitamin D levels, meanwhile did not find any significant evidence of blood pressure reduction by vitamin D supplementation. Considering that the included randomized controlled trials had small sample sizes and a short duration of follow-up, vitamin D supplementation might have positive effects on blood pressure control in the long term, especially in vitamin D-deficient individuals (98).

Serum vitamin D status might be linked to blood pressure through several mechanisms. The first possible mechanism for the association between a low concentration of 25(OH)D and HTN might be through the activation of the rennin–angiotensin system (RAS). It has been proved that the transcription renin gene could be inversely regulated by 1,25(OH)_2_D through a vitamin D receptor-mediated mechanism (99). As a result, vitamin D might play the role of a negative regulator to prevent the over-stimulation of the RAS. In fact, 1,25(OH)_2_D activates the vitamin D receptor which binds the cyclic adenosine monophosphate (CAMP)-response element-binding protein and blocks the renin gene promoter activity, thereby resulting in a decrease in renin secretion (100). Second, vitamin D might affect the cells of the vessel wall such as endothelial and vascular smooth muscle cells; all of these cells could express the vitamin D receptor as well as 1α-hydroxylase (101). Third, lower levels of 25(OH)D concentrations are associated with insulin resistance, and vitamin D supplementation may improve insulin production and insulin sensitivity (102). Insulin resistance has been suggested to be involved in the pathogenesis of hypertension (103). Fourth, vitamin D is indirectly associated with blood pressure due to the role of 25(OH)D in the regulation of calcium absorption (104) and in the maintenance of calcium homeostasis due to the interaction with parathyroid hormone (105). Fifth, 25(OH)D is proposed to have a role in reduction of free radicals local production, with positive effects on vascular health (106).

The current meta-analysis has some strengths and weaknesses. Our analysis included a large population of adults in both cohort and cross-sectional studies. The effect of several confounders was considered *via* a subgroup analysis. Dose–response analysis was also conducted. In addition, most eligible studies made adjustments for potential confounders including age, gender, BMI, and sampling time (season/month) or sun exposure. GRADE approach provided the certainty that serum vitamin D concentration is related to reduced odds of HTN and pre-HTN and may have a role in decreasing the risk of HTN. However, some limitations should be considered. The number of eligible studies that separately reported the relationship between blood vitamin D levels and hypertension in men and women was limited, so we could not provide appropriate estimates for males and females. More gender-specific studies are needed to obtain the relation between vitamin D and hypertension in males and females separately. In addition, studies were conducted on different age groups of adults and it could lead to heterogeneity because individuals with different age groups had different sun-exposure times and various rates of vitamin D synthesis due to differences in the capacity of the skin to synthesize vitamin D. Moreover, none of the cohort studies made an adjustment for the baseline vitamin D levels in their analyses.

In conclusion, this meta-analysis of epidemiologic studies disclosed that serum vitamin D concentration was inversely associated with risk of hypertension in adults, in a dose–response manner in both cohort and cross-sectional studies. The same association was found for pre-hypertension.

## Data Availability Statement

The original contributions presented in the study are included in the article/Supplementary Material, further inquiries can be directed to the corresponding author/s.

## Author Contributions

EM, ZH, and PS contributed in conception, design, statistical analyses, data interpretation, and manuscript drafting. All authors approved the final manuscript for submission.

## Funding

The financial support for this study comes from Isfahan University of Medical Sciences, Isfahan, Iran (no. 1400161). Isfahan University of Medical Sciences had no role in the design/conduct of the study, collection/analysis/interpretation of the data, and preparation/review/approval of the manuscript.

## Conflict of Interest

The authors declare that the research was conducted in the absence of any commercial or financial relationships that could be construed as a potential conflict of interest.

## Publisher's Note

All claims expressed in this article are solely those of the authors and do not necessarily represent those of their affiliated organizations, or those of the publisher, the editors and the reviewers. Any product that may be evaluated in this article, or claim that may be made by its manufacturer, is not guaranteed or endorsed by the publisher.
