# Supplementary material for: Serum Vitamin D Levels in Relation to Hypertension and Pre-hypertension in Adults: A Systematic Review and Dose–Response Meta-Analysis of Epidemiologic Studies
Source: Front Nutr. 2022 Mar 10;9:829307. doi: 10.3389/fnut.2022.829307 (PMC8961407; doi:10.3389/fnut.2022.829307)

**Online Supporting Material**

| **Supplemental Table 1.** MeSH and non-MeSH terms that were used in the systematic search | | |
| --- | --- | --- |
| **Database** | **Syntax** | **Results** |
| PubMed | (("Blood Pressures, High"[Mesh] OR "Blood Pressure, High"[Mesh] OR "High Blood Pressure"[Mesh] OR "High Blood Pressures"[Mesh] OR "Hypertension"[Mesh] OR "Hypertension" OR "High Blood Pressure" OR "Blood Pressure"[Mesh] OR "Blood Pressure" OR "Diastolic Pressure"[Mesh] OR "Diastolic Pressure" OR "Systolic Pressure"[Mesh] OR "Systolic Pressure" OR "hypertensive") AND ("Vitamin D"[Mesh] OR "Cholecalciferol" [Mesh] OR "Hydroxycholecalciferols " [Mesh] OR "Ergocalciferols" [Mesh] OR "25-Hydroxyvitamin D 2" [Mesh] OR "Dihydrotachysterol"[Mesh] OR "25(OH)D" OR "25-hydroxyvitamin D")) | 2608 |
| Web Of Sciences | ( "Blood Pressures, High"  OR  "Blood Pressure, High"  OR  "High Blood Pressure"  OR  "High Blood Pressures"  OR  "Hypertension"  OR  "High Blood Pressure"  OR  "Blood Pressure"  OR  "Diastolic Pressure"  OR  "Systolic Pressure"  OR  "hypertensive" )  AND  ( "Vitamin D"  OR  "Cholecalciferol"  OR  "Hydroxycholecalciferols "  OR  "Ergocalciferols"  OR  "25 Hydroxyvitamin D 2"  OR  "Dihydrotachysterol"  OR  "25(OH)D"  OR  "25-hydroxyvitamin D" ) | 4016 |
| Scopus | ( TITLE-ABS-KEY ( "Blood Pressures, High"  OR  "Blood Pressure, High"  OR  "High Blood Pressure"  OR  "High Blood Pressures"  OR  "Hypertension" OR  "Blood Pressure"  OR  "Diastolic Pressure"  OR  "Systolic Pressure"  OR  "hypertensive" )  AND  ( "Vitamin D"  OR  "Cholecalciferol"  OR  "Hydroxycholecalciferols "  OR  "Ergocalciferols"  OR  "25 Hydroxyvitamin D 2"  OR  "Dihydrotachysterol"  OR  "25(OH)D"  OR  "25-hydroxyvitamin D" ) ) | 8341 |
| Embase | ('Blood Pressures, High' :ti,ab,kw OR 'Blood Pressure, High':ti,ab,kw OR 'High Blood Pressures':ti,ab,kw OR 'High Blood Pressure':ti,ab,kw OR ' Blood Pressure':ti,ab,kw OR ' Hypertension':ti,ab,kw OR ' Diastolic Pressure':ti,ab,kw OR ' Systolic Pressure':ti,ab,kw OR ' hypertensive':ti,ab,kw) AND ('Vitamin D ':ti,ab,kw OR Cholecalciferol:ti,ab,kw OR Hydroxycholecalciferols:ti,ab,kw OR Ergocalciferols:ti,ab,kw OR '25 Hydroxyvitamin D 2':ti,ab,kw OR Dihydrotachysterol:ti,ab,kw OR 25(OH)D:ti,ab,kw OR 25-hydroxyvitamin D:ti,ab,kw ) | 5672 |

| **Supplemental Table 2. Preferred Reporting Items for Systematic Reviews and Meta- Analyses guideline (PRISMA).** | | | |
| --- | --- | --- | --- |
| **Section/topic** | **#** | **Checklist item** | **Reported on page #** |
| **TITLE** | | |  |
| Title | 1 | Identify the report as a systematic review, meta-analysis, or both. | √ Page1 |
| **ABSTRACT** | | |  |
| Structured summary | 2 | Provide a structured summary including, as applicable: background; objectives; data sources; study eligibility criteria, participants, and interventions; study appraisal and synthesis methods; results; limitations; conclusions and implications of key findings; systematic review registration number. | √ Page3 |
| **INTRODUCTION** | | |  |
| Rationale | 3 | Describe the rationale for the review in the context of what is already known. | √ Page4 |
| Objectives | 4 | Provide an explicit statement of questions being addressed with reference to participants, interventions, comparisons, outcomes, and study design (PICOS). | √ Page5 |
| **METHODS** | | |  |
| Protocol and registration | 5 | Indicate if a review protocol exists, if and where it can be accessed (e.g., Web address), and, if available, provide registration information including registration number. | √ Page5 |
| Eligibility criteria | 6 | Specify study characteristics (e.g., PICOS, length of follow-up) and report characteristics (e.g., years considered, language, publication status) used as criteria for eligibility, giving rationale. | √ Page6 |
| Information sources | 7 | Describe all information sources (e.g., databases with dates of coverage, contact with study authors to identify additional studies) in the search and date last searched. | √ Page6 |
| Search | 8 | Present full electronic search strategy for at least one database, including any limits used, such that it could be repeated. | √ Page6 |
| Study selection | 9 | State the process for selecting studies (i.e., screening, eligibility, included in systematic review, and, if applicable, included in the meta-analysis). | √ Page6-7 |
| Data collection process | 10 | Describe method of data extraction from reports (e.g., piloted forms, independently, in duplicate) and any processes for obtaining and confirming data from investigators. | √ Page7 |
| Data items | 11 | List and define all variables for which data were sought (e.g., PICOS, funding sources) and any assumptions and simplifications made. | √ Page7 |
| Risk of bias in individual studies | 12 | Describe methods used for assessing risk of bias of individual studies (including specification of whether this was done at the study or outcome level), and how this information is to be used in any data synthesis. | √ Page7 |
| Summary measures | 13 | State the principal summary measures (e.g., risk ratio, difference in means). | √ Page8 |
| Synthesis of results | 14 | Describe the methods of handling data and combining results of studies, if done, including measures of consistency (e.g., I^2^) for each meta-analysis. | √ Page8 |
| Risk of bias across studies | 15 | Specify any assessment of risk of bias that may affect the cumulative evidence (e.g., publication bias, selective reporting within studies). | √ Page8 |
| Additional analyses | 16 | Describe methods of additional analyses (e.g., sensitivity or subgroup analyses, meta-regression), if done, indicating which were pre-specified. | √ Page8 |
| **RESULTS** |  |  |  |
| Study selection | 17 | Give numbers of studies screened, assessed for eligibility, and included in the review, with reasons for exclusions at each stage, ideally with a flow diagram. | √ Page8, Fig 1 |
| Study characteristics | 18 | For each study, present characteristics for which data were extracted (e.g., study size, PICOS, follow-up period) and provide the citations. | √ Page9 |
| Risk of bias within studies | 19 | Present data on risk of bias of each study and, if available, any outcome level assessment (see item 12). | √ Page9, Table 1 |
| Results of individual studies | 20 | For all outcomes considered (benefits or harms), present, for each study: (a) simple summary data for each intervention group (b) effect estimates and confidence intervals, ideally with a forest plot. | √ Page9, Fig 2-5 |
| Synthesis of results | 21 | Present results of each meta-analysis done, including confidence intervals and measures of consistency. | √ Page 10-11 |
| Risk of bias across studies | 22 | Present results of any assessment of risk of bias across studies (see Item 15). | √ Page10-11 |
| Additional analysis | 23 | Give results of additional analyses, if done (e.g., sensitivity or subgroup analyses, meta-regression [see Item 16]). | √ Page11 |
| **DISCUSSION** |  |  |  |
| Summary of evidence | 24 | Summarize the main findings including the strength of evidence for each main outcome; consider their relevance to key groups (e.g., healthcare providers, users, and policy makers). | √ Page11 |
| Limitations | 25 | Discuss limitations at study and outcome level (e.g., risk of bias), and at review-level (e.g., incomplete retrieval of identified research, reporting bias). | √ Page14 |
| Conclusions | 26 | Provide a general interpretation of the results in the context of other evidence, and implications for future research. | √ Page14 |
| **FUNDING** |  |  |  |
| Funding | 27 | Describe sources of funding for the systematic review and other support (e.g., supply of data); role of funders for the systematic review. | √ Page15 |

Page 1 of 2

*From:*  Moher D, Liberati A, Tetzlaff J, Altman DG, The PRISMA Group (2009). Preferred Reporting Items for Systematic Reviews and Meta-Analyses: The PRISMA Statement. PLoS Med 6(6): e1000097. doi:10.1371/journal.pmed1000097

For more information, visit: **www.prisma-statement.org**.

Page 2 of 2

**Supplemental Table 3.** **Details of more relevant studies that were excluded.**

| **Ref.** | **First Author/ Year** | **Title of paper** | **Reason of exclusion from current systematic review and meta-analysis** |
| --- | --- | --- | --- |
| 1 | Nayak  (2016) | Evaluation of vitamin D relationship with type 2 diabetes and systolic blood pressure | Considered vitamin D deficiency as the outcome and hypertension as the exposure |
| 2 | Navaneethan  (2011) | Low 25-hydroxyvitamin D levels and mortality in non-dialysis-dependent CKD |  |
| 3 | Dao  (2011) | Low vitamin D among HIV-infected adults: prevalence of and risk factors for low vitamin D Levels in a cohort of HIV-infected adults and comparison to prevalence among adults in the US general population |  |
| 4 | Moberg  (2015) | Vitamin D deficiency and airflow limitation in the Baltimore Longitudinal Study of Ageing |  |
| 5 | Urea-Torres  (2011) | Association of kidney function, vitamin D deficiency, and circulating markers of mineral and bone disorders in CKD |  |
| 6 | Van Winden  (2019) | Low Bioactive Vitamin D Is Associated with Pregnancy-Induced Hypertension in a Cohort of Pregnant HIV-Infected Women Sampled Over a 23-Year Period | Reported the relation between levels of serum 25-hydroxyvitamin D with Pregnancy-Induced hypertension |
| 7 | Hossain  (2011) | High prevalence of vitamin D deficiency in Pakistani mothers and their newborns |  |
| 8 | Abedi  (2014) | The relationship of serum vitamin D with pre-eclampsia in the Iranian women |  |
| 9 | Baca  (2016) | Low maternal 25-hydroxyvitamin D concentration increases the risk of severe and mild preeclampsia |  |
| 10 | Barebring  (2016) | Preeclampsia and Blood Pressure Trajectory during Pregnancy in Relation to Vitamin D Status |  |
| 11 | Cabral  (2016) | Vitamin D levels and cardiometabolic risk factors in Portuguese adolescents | Reported the relation between levels of serum 25-hydroxyvitamin D with hypertension in children and adolescents |
| 12 | Kao  (2015) | Low vitamin D is associated with hypertension in paediatric obesity |  |
| 13 | Lee  (2013) | Serum 25-hydroxyvitamin D levels, obesity and the metabolic syndrome among Korean children |  |
| 14 | Nam  (2014) | 25-Hydroxyvitamin D insufficiency is associated with cardiometabolic risk in Korean adolescents: the 2008-2009 Korea National Health and Nutrition Examination Survey (KNHANES) |  |
| 15 | Pacifico  (2011) | Low 25(OH)D-3 levels are associated with total adiposity, metabolic syndrome, and hypertension in Caucasian children and adolescents |  |
| 16 | Reis  (2009) | Vitamin D status and cardiometabolic risk factors in the United States adolescent population |  |
| 17 | Chiang  (2017) | 25-Hydroxyvitamin D and blood pressure: a plateau effect in adults with African ancestry living at different latitudes | Reported the relation between levels of serum 25-Hydroxyvitamin D with blood pressure as a continuous variable |
| 18 | Bhandari  (2011) | 25-Hydroxyvitamin D Levels and Hypertension  Rates | Considered severity of hypertension as the Outcome |
| 19 | Alagacone  (2020) | The association between vitamin D deficiency and the risk of resistant hypertension |  |
| 20 | Sypniewska  (2014) | 25-hydroxyvitamin D, biomarkers of endothelial dysfunction and subclinical organ damage in adults with hypertension |  |
| 21 | Sebati  (2020) | The Relationship between Low 25-Hydroxyvitamin D and Cardio-Metabolic Risk Factors among Ellisras Young Adults | Reported standard regression coefficient |
| 22 | Alsayed  (2007) | Serum 25-hydroxyvitamin D concentrations and metabolic syndrome in Egyptian men | Reported correlation coefficient for the relationship |
| 23 | Ashok  (2017) | Associations of vitamin D with metabolic syndrome components in Indian urban middle-aged women |  |
| 24 | Alkhatatbeh  (2019) | Association of 25-hydroxyvitamin D with HDL-cholesterol and other cardiovascular risk biomarkers in subjects with non-cardiac chest pain |  |

**REFERENCES:**

1. Nayak SB, Ramnanansingh TG. Evaluation of vitamin D relationship with type 2 diabetes and systolic blood pressure. BMJ open diabetes research & care. 2016;4(1):e000285.
2. Navaneethan SD, Schold JD, Arrigain S, Jolly SE, Jain A, Schreiber MJ, Jr., et al. Low 25-hydroxyvitamin D levels and mortality in non-dialysis-dependent CKD. American journal of kidney diseases : the official journal of the National Kidney Foundation. 2011;58(4):536-43.
3. Dao CN, Patel P, Overton ET, Rhame F, Pals SL, Johnson C, et al. Low vitamin D among HIV-infected adults: prevalence of and risk factors for low vitamin D Levels in a cohort of HIV-infected adults and comparison to prevalence among adults in the US general population. Clinical infectious diseases : an official publication of the Infectious Diseases Society of America. 2011;52(3):396-405.
4. Moberg M, Elango P, Ferrucci L, Spruit MA, Wouters EF, Rutten EP. Vitamin D deficiency and airflow limitation in the Baltimore Longitudinal Study of Ageing. European journal of clinical investigation. 2015;45(9):955-63.
5. Ureña-Torres P, Metzger M, Haymann JP, Karras A, Boffa JJ, Flamant M, et al. Association of kidney function, vitamin D deficiency, and circulating markers of mineral and bone disorders in CKD. American journal of kidney diseases : the official journal of the National Kidney Foundation. 2011;58(4):544-53.
6. Van Winden KR, Bearden A, Kono N, Frederick T, Operskalski E, Stek A, et al. Low Bioactive Vitamin D Is Associated with Pregnancy-Induced Hypertension in a Cohort of Pregnant HIV-Infected Women Sampled Over a 23-Year Period. American journal of perinatology. 2020;37(14):1446-54.
7. Hossain N, Khanani R, Hussain-Kanani F, Shah T, Arif S, Pal L. High prevalence of vitamin D deficiency in Pakistani mothers and their newborns. International journal of gynaecology and obstetrics: the official organ of the International Federation of Gynaecology and Obstetrics. 2011;112(3):229-33.
8. Abedi P, Mohaghegh Z, Afshary P, Latifi M. The relationship of serum vitamin D with pre-eclampsia in the Iranian women. Maternal & child nutrition. 2014;10(2):206-12.
9. Baca KM, Simhan HN, Platt RW, Bodnar LM. Low maternal 25-hydroxyvitamin D concentration increases the risk of severe and mild preeclampsia. Annals of epidemiology. 2016;26(12):853-7.e1.
10. Bärebring L, Bullarbo M, Glantz A, Leu Agelii M, Jagner Å, Ellis J, et al. Preeclampsia and Blood Pressure Trajectory during Pregnancy in Relation to Vitamin D Status. PloS one. 2016;11(3):e0152198.
11. Cabral M, Araújo J, Teixeira J, Barros H, Martins S, Guimarães JT, et al. Vitamin D levels and cardiometabolic risk factors in Portuguese adolescents. International journal of cardiology. 2016;220:501-7.
12. Kao KT, Abidi N, Ranasinha S, Brown J, Rodda C, McCallum Z, et al. Low vitamin D is associated with hypertension in paediatric obesity. Journal of paediatrics and child health. 2015;51(12):1207-13.
13. Lee SH, Kim SM, Park HS, Choi KM, Cho GJ, Ko BJ, et al. Serum 25-hydroxyvitamin D levels, obesity and the metabolic syndrome among Korean children. Nutrition, metabolism, and cardiovascular diseases : NMCD. 2013;23(8):785-91.
14. 25-Hydroxyvitamin D insufficiency is associated with cardiometabolic risk in Korean adolescents: the 2008-2009 Korea National Health and Nutrition Examination Survey (KNHANES)
15. Pacifico L, Anania C, Osborn JF, Ferraro F, Bonci E, Olivero E, et al. Low 25(OH)D3 levels are associated with total adiposity, metabolic syndrome, and hypertension in Caucasian children and adolescents. European journal of endocrinology. 2011;165(4):603-11.
16. Reis JP, von Mühlen D, Miller ER, 3rd, Michos ED, Appel LJ. Vitamin D status and cardiometabolic risk factors in the United States adolescent population. Pediatrics. 2009;124(3):e371-9.
17. Chiang D, Kramer H, Luke A, Cooper R, Aloia J, Bovet P, et al. 25-Hydroxyvitamin D and blood pressure: a plateau effect in adults with African ancestry living at different latitudes. Journal of hypertension. 2017;35(5):968-74.
18. Bhandari SK, Pashayan S, Liu IL, Rasgon SA, Kujubu DA, Tom TY, et al. 25-hydroxyvitamin D levels and hypertension rates. Journal of clinical hypertension (Greenwich, Conn). 2011;13(3):170-7.
19. Alagacone S, Verga E, Verdolini R, Saifullah SM. The association between vitamin D deficiency and the risk of resistant hypertension. Clinical and experimental hypertension (New York, NY : 1993). 2020;42(2):177-80.
20. Sypniewska G, Pollak J, Strozecki P, Camil F, Kretowicz M, Janikowski G, et al. 25-hydroxyvitamin D, biomarkers of endothelial dysfunction and subclinical organ damage in adults with hypertension. American journal of hypertension. 2014;27(1):114-21.
21. Sebati B, Monyeki K, Monyeki S. The Relationship between Low 25-Hydroxyvitamin D and Cardio-Metabolic Risk Factors among Ellisras Young Adults. International journal of environmental research and public health. 2020;17(20).
22. Alsayed A, Gad A, Azab A. Serum 25-hydroxyvitamin D concentrations and metabolic syndrome in Egyptian men. Journal of Medical Sciences. 2007;7(5):850-4.
23. Ashok P, Balsubramanian B, Joshi S, Kharche JS, Vaidya SM. Associations of vitamin D with metabolic syndrome components in Indian urban middle-aged women. National Journal of Physiology, Pharmacy and Pharmacology. 2017;7(5):497-500.
24. Alkhatatbeh MJ, Amara NA, Abdul-Razzak KK. Association of 25-hydroxyvitamin D with HDL-cholesterol and other cardiovascular risk biomarkers in subjects with non-cardiac chest pain. Lipids in health and disease. 2019;18(1):27.

**Supplemental Table 4**. Details of quality assessment of included studies in the systematic review and meta-analysis based on Newcastle-Ottawa Scale^1^.

1. Cohort and nested case-control studies.

|  | SELECTION | | | | COMPARABILITY | OUTCOME | | |  |
| --- | --- | --- | --- | --- | --- | --- | --- | --- | --- |
|  | Representativeness of the exposed cohort | Selection of the non-exposed cohort | Ascertainment of exposure | Demonstration that outcome of interest was not present at start of study | Comparability of cohorts on the basis of the design or analysis | Assessment of outcome | Was follow-up long enough (5year)for outcomes to occur | Adequacy of follow up of cohorts | Total score |
| Forman et al, 2008 |  | * | * | * | * | * | * | * | 7 |
| Anderson et al, 2010 | * | * | * | * |  | * | * |  | 6 |
| Forman et al, 2007 | * | * | * | * | * | * | * |  | 7 |
| Jorde et al, 2010 | * | * | * |  | * | * | * | * | 7 |
| Ke et al, 2013 |  | * | * |  | * | * | * | * | 6 |
| Margolis et al, 2012 |  | * | * | * | * | * | * | * | 7 |
| Van Ballegooijen et al, 2015 | * | * | * | * | ** | * | * | * | 9 |
| van Ballegooijen et al, 2014 | * | * | * | * | * | * | * | * | 8 |
| Wang et al, 2013 |  | * | * | * | * |  | * | * | 6 |
| Gagnon et al, 2012 | * | * | * | * | * | * | * | * | 8 |
| Skaaby et al, 2012 | * | * | * | * | ** | * | * | * | 9 |

1. Cross-sectional studies.

|  | SELECTION | | | | COMPARABILITY | OUTCOME | |  |  |  |  |
| --- | --- | --- | --- | --- | --- | --- | --- | --- | --- | --- | --- |
|  | Representativeness of the sample | Sample size | Non-respondents | Ascertainment of the exposure (risk factor) | Comparability of subjects in different outcome groups | Assessment of outcome | Statistical test | Total score |  |  |  |
| Lee et al, 2019 | | * | * |  | ** | * | ** | * | 8 |  |  |
| Vitezova et al, 2015 | | * | * | * | ** | * | ** | * | 9 |  |  |
| Chon et al 2014 | | * | * | * | ** | * | ** | * | 9 |  |  |
| Maki et al, 2012 | | | * | * | * | ** |  | ** | * | 8 |  |
| Mansouri et al, 2018 | | |  | * | * | ** |  | ** | * | 7 |  |
| Schmitt et al, 2018 | | |  |  | * | ** |  | ** | * | 6 |  |
| Bea et al, 2015 | | | * | * |  | ** |  | ** | * | 7 |  |
| Kim et al, 2012 | | | * | * | * | ** | ** | ** | * | 10 |  |
| Majumdar et al, 2011 | | | * |  |  | ** | * |  | * | 5 |  |
| Akter et al, 2017 | | |  |  | * | ** | * | ** | * | 7 |  |
| Barceló et al, 2013 | | |  |  |  | ** | * |  | * | 4 |  |
| Mitri et al, 2014 | | | * |  | * | ** | ** | ** | * | 9 |  |
| Ahmadi et al, 2016 | | |  |  |  | ** |  | ** | * | 5 |  |
| Brock et al, 2011 | | | * | * |  | ** | * | * | * | 7 |  |
| Burgaz et al, 2011 | | |  | * | * | ** | * | ** | * | 8 |  |
| Dorjgochoo et al, 2012 | | | * |  | * | ** | ** | ** | * | 9 |  |
| García‐Carrasco et al, 2019 | | |  |  | * | ** |  | ** | * | 6 |  |
| Hidru et al, 2019 | | |  |  | * | ** | * | ** | * | 7 |  |
| Hirani et al, 2014 | | | * |  | * | ** |  | ** | * | 7 |  |
| Hyppo¨nen et al, 2008 | | | | * | * | * | ** | ** | ** | * | 10 |
| Joukar et al, 2020 | | | | * | * |  | ** | ** | ** | * | 9 |
| Ke et al, 2013 | | | |  | * | * | ** | * | ** | * | 8 |
| Kim et al, 2016 | | | | * | * | * | ** | ** | ** | * | 10 |
| Kim et al, 2016 | | | | * | * | * | ** | ** | ** | * | 10 |
| Kim et al, 2015 | | | | * | * | * | ** | ** | ** | * | 10 |
| Kim et al, 2015 | | | |  |  |  | ** | ** | ** | * | 7 |
| Kim et al, 2010 | | | | * | * | * | ** | ** | ** | * | 10 |
| Kwak et al, 2020 | | | | * | * | * | ** | * | ** | * | 9 |
| Lertratanakul et al, 2014 | | | |  | * | * |  | ** | ** | * | 7 |
| Liu t al, 2020 | | | | * |  |  | ** | * | ** | * | 7 |
| Peng et al, 2016 | | | | * |  | * | ** |  | ** | * | 7 |
| Reis et al, 2007 | | | | * |  | * | ** | * | ** | * | 8 |
| Shen et al, 2020 | | | | * | * | * | ** |  |  | * | 6 |
| Snijder et al, 2007 | | | | * |  | * | ** | * | ** | * | 8 |
| Song et al, 2013 | | | |  | * | * | ** | * | ** | * | 8 |
| Steinvil et al, 2011 | | | | * | * |  | ** |  | ** | * | 7 |
| Martins et al, 2007 | | | | * | * | * | ** |  | ** | * | 8 |
| Khader et al, 2011 | | | | * |  |  | ** | ** | ** | * | 8 |
| Jeenduang et al, 2020 | | | | * | * | * | ** |  | ** | * | 8 |
| Gupta et al, 2012 | | | | * |  | * | ** | * | ** | * | 8 |
| Gupta et al, 2012 | | | | * |  | * | ** | * | ** | * | 8 |
| Gupta et al, 2011 | | | | * |  | * |  | * | ** | * | 6 |
| Esteghamati et al, 2014 | | | | * | * | * | ** | * | ** | * | 9 |
| Contreras-Manzano et al, 2019 | | | | * |  |  | ** | * | ** | * | 7 |
| Caro et al, 2012 | | | |  |  | * | ** | ** | ** | * | 8 |
| Zhao et al, 2010 | | | * | * | * | ** | * | ** | * | 9 |  |
| Sumriddetchkajorn et al, 2012 | | | * | * |  |  | * |  | * | 4 |  |
| Shin et al, 2015 | | | * | * | * | ** | * | ** | * | 9 |  |
| Pannu et al, 2017 | | | * |  | * | ** | ** | ** | * | 9 |  |
| Li et al, 2012 | | |  |  | * | ** | * | ** | * | 7 |  |
| Kwak et al, 2019 | | | * | * | * | ** | * | ** | * | 9 |  |
| Dong et al, 2014 | | |  |  | * | ** |  | ** | * | 6 |  |
| Chen et al, 2015 | | |  | * | * | ** | ** | ** | * | 9 |  |
| Chen et al, 2019 | | | * | * | * | ** |  | ** | * | 8 |  |
| Sabanayagam et al, 2012 | | | * | * | * | ** | * | ** | * | 9 |  |
| Vacek et al, 2012 | | | * | * | * | ** |  | ** | * | 8 |  |
| Mateus-Hamdan et al, 2013 | | |  |  | * | ** | * | ** | * | 7 |  |
| Ford et al, 2005 | | | * |  |  | ** | * | ** | * | 7 |  |
| Muldowney et al, 2011 | | |  |  | * | ** | * | ** | * | 7 |  |
| Piantanida et al, 2017 | | |  |  | * | ** |  | ** | * | 6 |  |

^1^Wells GA, Shea B, O’Connell D, Peterson J, Welch V, Tugwell P. The Newcastle-Ottawa Scale (NOS) for Assessing the Quality of Nonrandomised Studies in Meta-Analyses. Available from: http://www.ohri.ca/programs/clinical_epidemiology/oxford.asp

***Supplementary Table 5. GRADE evidence profile for serum vitamin D concentration in relation to Hypertension***

| **Certainty assessment** | | | | | | | **№ of patients** | | **Effect** | | **Certainty** | **Importance** |
| --- | --- | --- | --- | --- | --- | --- | --- | --- | --- | --- | --- | --- |
| **№ of studies** | **Study design** | **Risk of bias** | **Inconsistency** | **Indirectness** | **Imprecision** | **Other considerations** | **Participants** | **Cases** | **Relative (95% CI)** | **Absolute (95% CI)** |  |  |
| 11 | Cohort studies | Not serious^a^ | Not serious^b^ | Not serious | Serious^c^ | Dose response gradient | 66757 | 26222  (39.3%) | **RR 0.84** (0.73 to 0.96) | **63 fewer per 1,000** (from 106 fewer to 16 fewer) | ⨁⨁⨁⨁ High | IMPORTANT |
| 56 | Cross-sectional studies | Not serious^d^ | Not serious^e^ | Not serious | Not serious | Dose response gradient | 248657 | 54848  (22.1%) | **OR 0.84 (0.79 to 0.90)** | **29 fewer per 1,000** (from 38 fewer to 18 fewer) | ⨁⨁⨁⨁ High | IMPORTANT |

**CI:** confidence interval; **RR:** risk ratio

*a. Serious risk of bias; since, our quality assessment was based on Newcastle-Ottawa Scale. However, the effect size in the subgroup of studies with high quality was the same with the main analysis (RR: 0.88 (0.78, 0.99); n=9). Not downgraded.*

b. *Serious inconsistency since I^2^=64%. However,* excluding one study of Anderson et al. removed the observed heterogeneity (I^2^= 24.2%, P= 0.21), without significant changing in overall estimate (RR=0.89; 95%CI: 0.81, 0.99). *Not downgraded.*

*c. Serious imprecision; since, 95%CI contained minimal value of 0.75.*

d. *Serious risk of bias; since, our quality assessment was based on Newcastle-Ottawa Scale. However, the effect size in the subgroup of studies with high quality was the same with the main analysis (RR: 0.82 (0.76, 0.89); n=36). Not downgraded.*

e. *Serious inconsistency since I^2^=67.5%. However, value of I^2^ was <50% in the subgroup of studies conducted female participants, significant, direction, and magnitude of the effect remained unchanged (RR: 0.82 (0.77, 0.87); n=17, I^2^=0.0%). Not downgraded.*

***Supplementary Table 6. GRADE evidence profile for serum vitamin D concentration in relation to Pre-Hypertension***

| **Certainty assessment** | | | | | | | **№ of patients** | | **Effect** | | **Certainty** | **Importance** |
| --- | --- | --- | --- | --- | --- | --- | --- | --- | --- | --- | --- | --- |
| **№ of studies** | **Study design** | **Risk of bias** | **Inconsistency** | **Indirectness** | **Imprecision** | **Other considerations** | **Participants** | **Cases** | **Relative (95% CI)** | **Absolute (95% CI)** |  |  |
| 7 | Cross-sectional studies | Not serious^a^ | Not serious | Not serious | Serious^b^ | None | 21242 | 6654 (31.3%) | **OR 0.75** (0.68 to 0.83) | **58 fewer per 1,000** (from 77 fewer to 39 fewer) | ⨁⨁⨁◯ Moderate | IMPORTANT |

*a. Serious risk of bias; since, our quality assessment was based on Newcastle-Ottawa Scale. However, the effect size in the subgroup of studies with high quality was the same with the main analysis (RR: 0.78 (0.69, 0.87); n=8). Not downgraded.*

*b. Serious imprecision; since, 95%CI contained minimal value of 0.75.*

**Supplemental Table 7**. STATA codes used to examine the association between serum vitamin D levels and high blood pressure in adults.

| **Meta-analysis of highest versus lowest vitamin D level in relation to hypertension in prospective studies:** |
| --- |
| 1. metan LogOR SELogOR if Outcome_definition!=1 & Design2status==2, label(namevar=Author, yearvar=Year) sortby(Year) random eform 2. metan LogOR SELogOR if Outcome_definition!=1 & Design2status==2, label(namevar=Author, yearvar=Year) sortby(Year) by(Sex) random eform 3. metan LogOR SELogOR if Outcome_definition!=1 & Design2status==2, label(namevar=Author, yearvar=Year) sortby(Year) by(Sex) fixed ef orm 4. metan LogOR SELogOR if Outcome_definition!=1 & Design2status==2, label(namevar=Author, yearvar=Year) sortby(Year) by(comparison) r andom eform 5. metan LogOR SELogOR if Outcome_definition!=1 & Design2status==2, label(namevar=Author, yearvar=Year) sortby(Year) by(comparison) fixed eform 6. metan LogOR SELogOR if Outcome_definition!=1 & Design2status==2, label(namevar=Author, yearvar=Year) sortby(Year) by(Adjust_Time_of_blood_draw) random eform 7. metan LogOR SELogOR if Outcome_definition!=1 & Design2status==2, label(namevar=Author, yearvar=Year) sortby(Year) by(Adjust_Time_o f_blood_draw) fixed eform 8. metan LogOR SELogOR if Outcome_definition!=1 & Design2status==2, label(namevar=Author, yearvar=Year) sortby(Year) by(Adjust_Age_Se x_BMI) random eform 9. metan LogOR SELogOR if Outcome_definition!=1 & Design2status==2, label(namevar=Author, yearvar=Year) sortby(Year) by(Adjust_Age_Se x_BMI) fixed eform 10. metan LogOR SELogOR if Outcome_definition!=1 & Design2status==2, label(namevar=Author, yearvar=Year) sortby(Year) by(Representative) random eform 11. metan LogOR SELogOR if Outcome_definition!=1 & Design2status==2, label(namevar=Author, yearvar=Year) sortby(Year) by(Representative) fixed eform 12. metan LogOR SELogOR if Outcome_definition!=1 & Design2status==2, label(namevar=Author, yearvar=Year) sortby(Year) by(Qualitystatus) random eform 13. metan LogOR SELogOR if Outcome_definition!=1 & Design2status==2, label(namevar=Author, yearvar=Year) sortby(Year) by(Qualitystatus) fixed eform 14. metan LogOR SELogOR if Outcome_definition!=1 & Design2status==2 & ID_STUDY!=18, label(namevar=Author, yearvar=Year) sortby(Year) random eform 15. metaninf LogOR SELogOR if Outcome_definition!=1 & Design2status==2, label(namevar=Author, yearvar=Year) random 16. metabias LogOR SELogOR if Outcome_definition!=1 & Design2status==2, graph(begg) |
| **Dose-response meta-analysis of serum vitamin D and risk of hypertension in prospective studies:** |
| 1. metan LnRR SELnRR, label(namevar=Author, yearvar=Year) sortby(Year) random eform |
| **Meta-analysis of highest versus lowest vitamin D level in relation to hypertension in cross-sectional studies:** |
| 1. metan LogOR SELogOR if Outcome_definition!=1 & Design2status==1, label(namevar=Author, yearvar=Year) sortby(Year) random eform 2. metan LogOR SELogOR if Outcome_definition!=1 & Design2status==1, label(namevar=Author, yearvar=Year) sortby(Year) by(AsianVSNonAsian) random eform 3. metan LogOR SELogOR if Outcome_definition!=1 & Design2status==1, label(namevar=Author, yearvar=Year) sortby(Year) by(AsianVSNonAsian) fixed eform 4. metan LogOR SELogOR if Outcome_definition!=1 & Design2status==1, label(namevar=Author, yearvar=Year) sortby(Year) by(DevelopingStatus) random eform 5. metan LogOR SELogOR if Outcome_definition!=1 & Design2status==1, label(namevar=Author, yearvar=Year) sortby(Year) by(DevelopingStatus) fixed eform 6. metan LogOR SELogOR if Outcome_definition!=1 & Design2status==1, label(namevar=Author, yearvar=Year) sortby(Year) by(Sex) random eform 7. metan LogOR SELogOR if Outcome_definition!=1 & Design2status==1, label(namevar=Author, yearvar=Year) sortby(Year) by(Sex) fixed eform 8. metan LogOR SELogOR if Outcome_definition!=1 & Design2status==1, label(namevar=Author, yearvar=Year) sortby(Year) by(comparison) random eform 9. metan LogOR SELogOR if Outcome_definition!=1 & Design2status==1, label(namevar=Author, yearvar=Year) sortby(Year) by(comparison) fixed eform 10. metan LogOR SELogOR if Outcome_definition!=1 & Design2status==1, label(namevar=Author, yearvar=Year) sortby(Year) by(Outcome_definition) random eform 11. metan LogOR SELogOR if Outcome_definition!=1 & Design2status==1, label(namevar=Author, yearvar=Year) sortby(Year) by(Outcome_definition) fixed eform 12. metan LogOR SELogOR if Outcome_definition!=1 & Design2status==1, label(namevar=Author, yearvar=Year) sortby(Year) by(subject) random eform 13. metan LogOR SELogOR if Outcome_definition!=1 & Design2status==1, label(namevar=Author, yearvar=Year) sortby(Year) by(subject) fixed eform 14. metan LogOR SELogOR if Outcome_definition!=1 & Design2status==1, label(namevar=Author, yearvar=Year) sortby(Year) by(Adjust_Time_of_blood_draw) random eform 15. metan LogOR SELogOR if Outcome_definition!=1 & Design2status==1, label(namevar=Author, yearvar=Year) sortby(Year) by(Adjust_Time_of_blood_draw) fixed eform 16. metan LogOR SELogOR if Outcome_definition!=1 & Design2status==1, label(namevar=Author, yearvar=Year) sortby(Year) by(Adjust_Age_Sex_BMI) random eform 17. metan LogOR SELogOR if Outcome_definition!=1 & Design2status==1, label(namevar=Author, yearvar=Year) sortby(Year) by(Adjust_Age_Sex_BMI) fixed eform 18. metan LogOR SELogOR if Outcome_definition!=1 & Design2status==1, label(namevar=Author, yearvar=Year) sortby(Year) by(Representative) random eform 19. metan LogOR SELogOR if Outcome_definition!=1 & Design2status==1, label(namevar=Author, yearvar=Year) sortby(Year) by(Representative) fixed eform 20. metan LogOR SELogOR if Outcome_definition!=1 & Design2status==1, label(namevar=Author, yearvar=Year) sortby(Year) by(Qualitystatus) random eform 21. metan LogOR SELogOR if Outcome_definition!=1 & Design2status==1, label(namevar=Author, yearvar=Year) sortby(Year) by(Qualitystatus) fixed eform 22. metaninf LogOR SELogOR if Outcome_definition!=1 & Design2status==1, label(namevar=Author, yearvar=Year) random 23. metabias LogOR SELogOR if Outcome_definition!=1 & Design2status==1, graph(begg) |
|  |
| **Dose-response meta-analysis of serum vitamin D and risk of hypertension in cross-sectional studies:** |
| 1. metan LogRR SELogRR, label(namevar=Author, yearvar=Year) sortby(Year) random eform 2. metan LogRR SELogRR, label(namevar=Author, yearvar=Year) sortby(Year) random eform |
| **Meta-analysis of highest versus lowest vitamin D level in relation to pre-hypertension in cross-sectional studies:** |
| 1. metan LogOR SELogOR if Outcome_definition==1 & Design2status==1, label(namevar=Author, yearvar=Year) sortby(Year) fixed eform 2. metaninf LogOR SELogOR if Outcome_definition==1 & Design2status==1, label(namevar=Author, yearvar=Year) random 3. metabias LogOR SELogOR if Outcome_definition==1 & Design2status==1, graph(begg) |

**Supplemental Figure 1:** Funnel plots of begg's test for publication biases.

1. Funnel plot for publication bias in prospective studies.
2. Funnel plot for publication bias in cross-sectional studies.
3. Funnel plot for publication bias for pre-HTN.

**Supplemental Figure 2:** Linear dose-response meta-analysis of serum vitamin D and odds of HTN in cross-sectional studies.


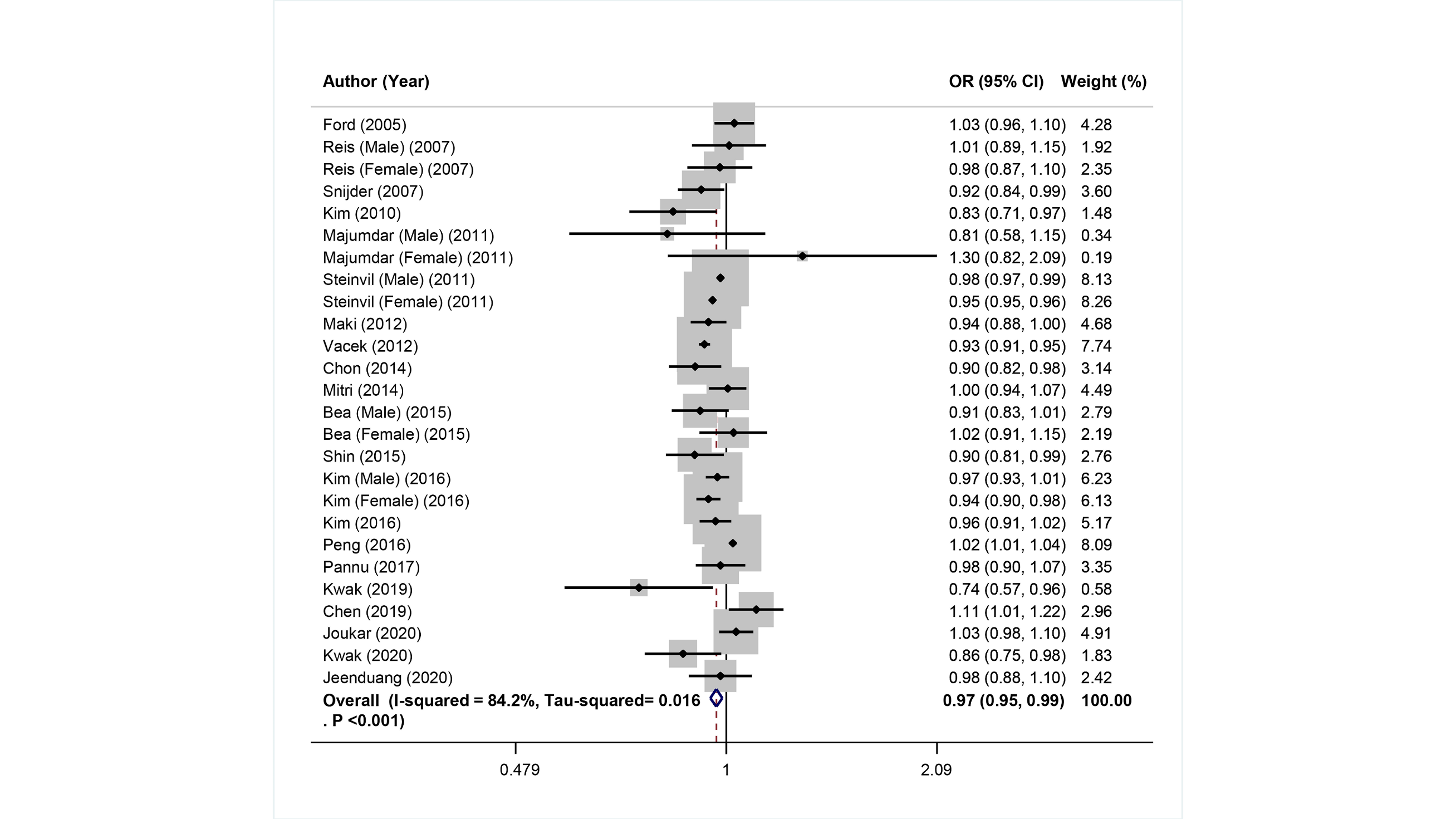


**Supplemental Figure 3:** Non-Linear dose-response meta-analysis of serum vitamin D and odds of HTN in cross-sectional studies.

**Supplemental Figure 4:** Linear dose-response meta-analysis of serum vitamin D and odds of HTN in cross-sectional studies with representative population.


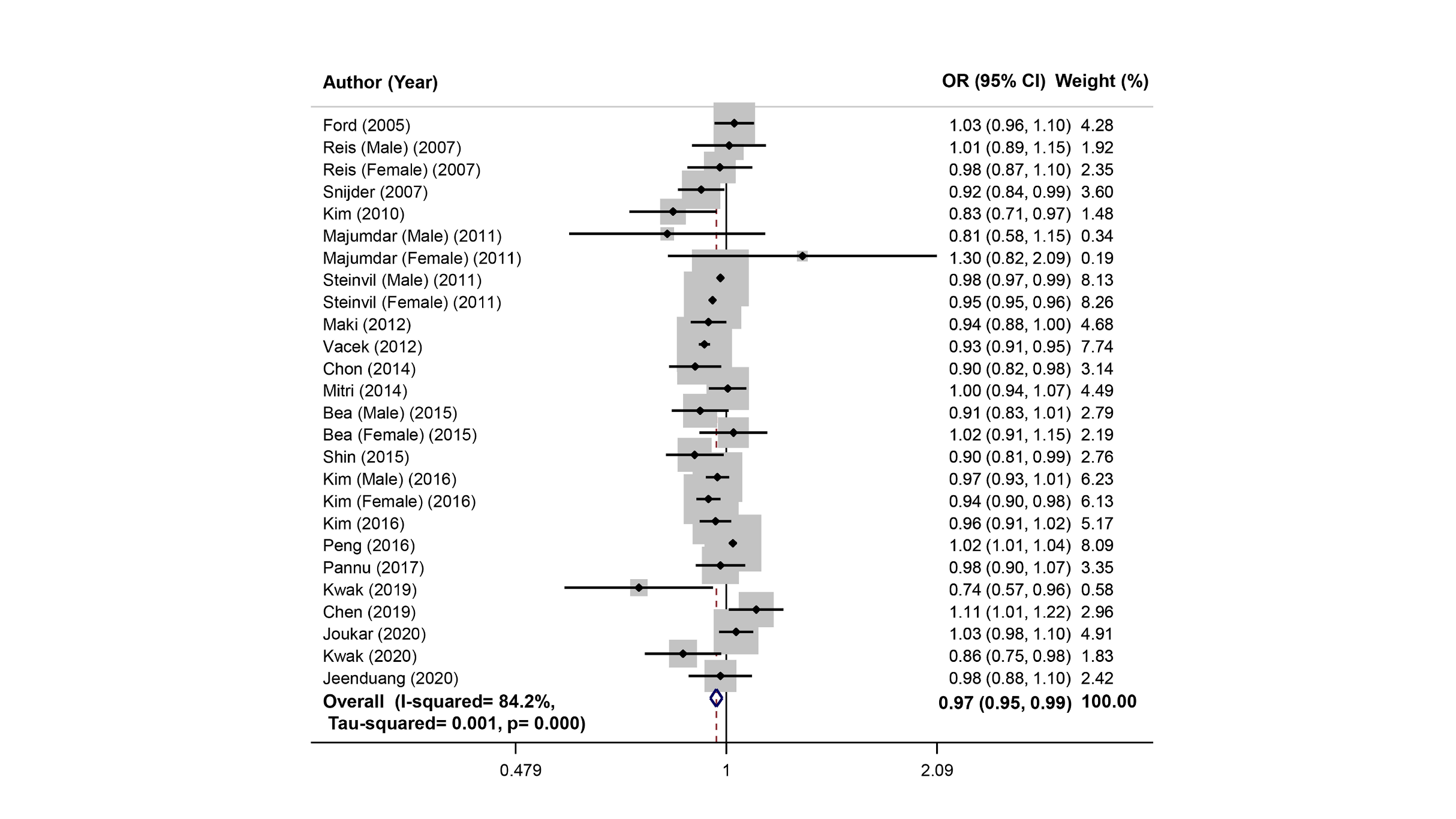


**Supplemental Figure 5:** Non-Linear dose-response meta-analysis of serum vitamin D and odds of HTN in cross-sectional studies with representative population.

**Supplemental Figure 6:** Forest plot cross-sectional studies that examined the association between highest vs. lowest level of serum vitamin D and risk of pre-hypertension.


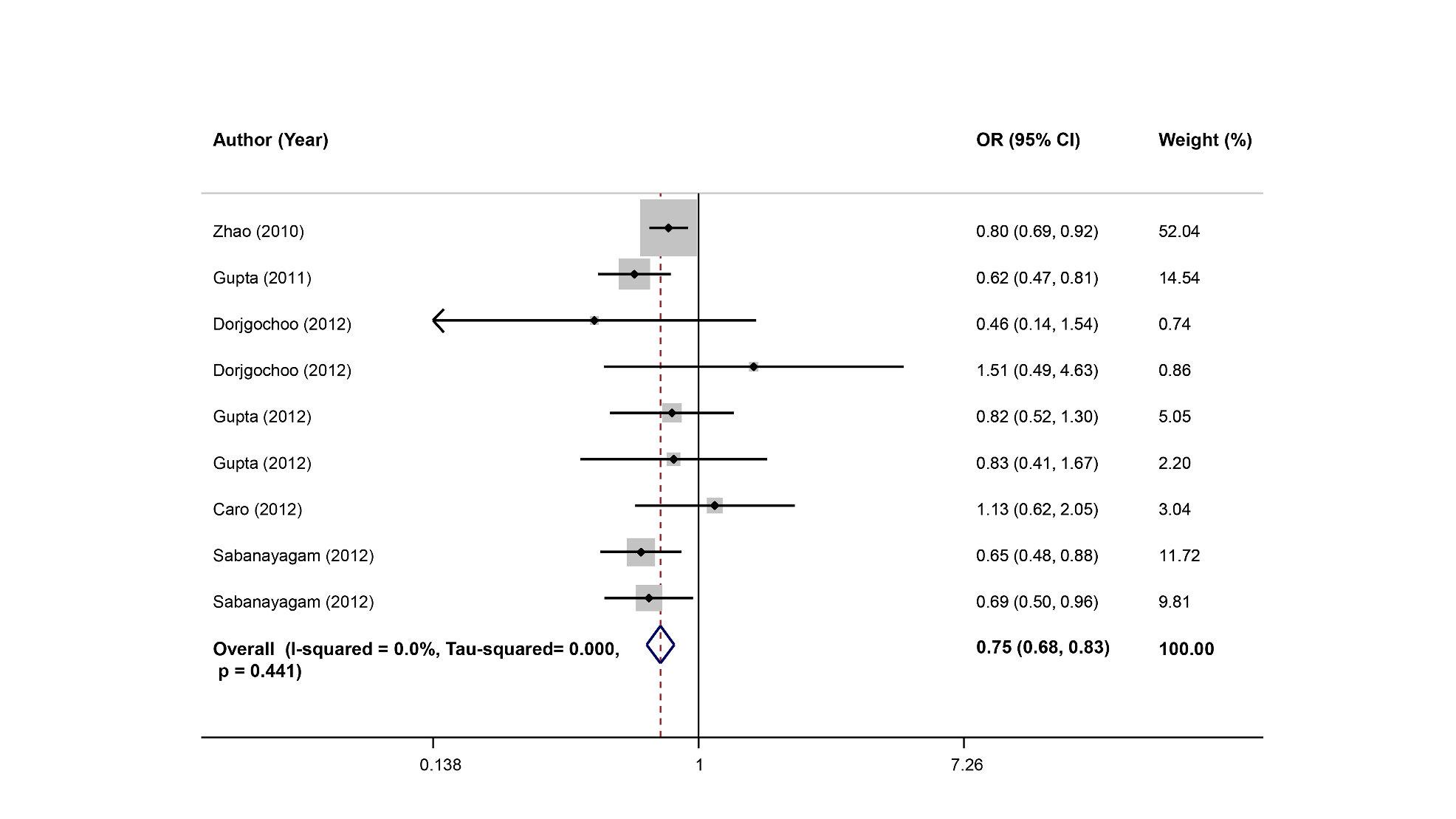

Supplement: Supplementary file 1 [file Data_Sheet_1.docx]
