# Supplementary material for: Serum Vitamin D Levels in Relation to Hypertension and Pre-hypertension in Adults: A Systematic Review and Dose–Response Meta-Analysis of Epidemiologic Studies
Source: Front Nutr. 2022 Mar 10;9:829307. doi: 10.3389/fnut.2022.829307 (PMC8961407; doi:10.3389/fnut.2022.829307)
Supplement: Supplementary file 2 [file Data_Sheet_2.docx]

- Findings of observational studies that evaluated the association of serum vitamin D status and high blood pressure were contradictory.
- This meta-analysis revealed an inverse association between serum vitamin D concentrations and risk of hypertension in adults, in both prospective cohort and cross-sectional studies. This inverse association was independent of hypertension definition (BP≥140/90 vs. ≥130/85 mmHg).
- Dose–response analysis showed significant linear and non-linear relationships between serum vitamin D and risk of hypertension.
